# Interactions between Small Inorganic Ions and Uncharged Monolayers on the Water/Air Interface

**DOI:** 10.1021/acs.jpcb.2c08019

**Published:** 2023-03-17

**Authors:** Boyan Peychev, Radomir I. Slavchov

**Affiliations:** Queen Mary University of London, School of Engineering and Materials Science, Mile End Road, London E1 4NS, United Kingdom

## Abstract

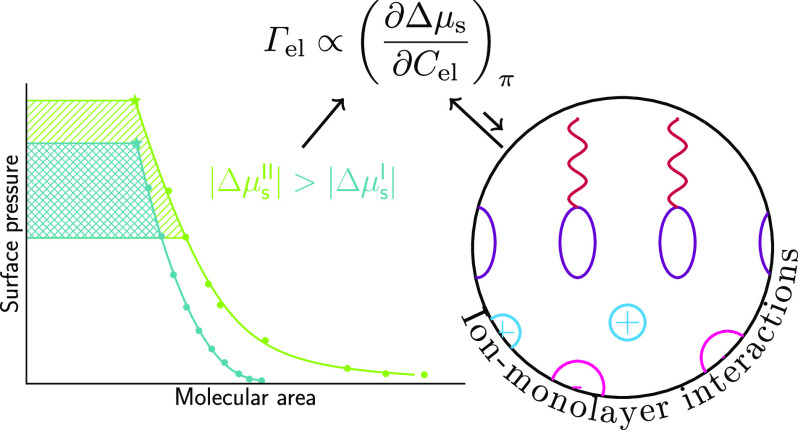

The interaction of several simple electrolytes with uncharged insoluble monolayers is studied on the basis of tensiometric and potentiometric data for the surface electrolyte solution|air. The induced adsorption of electrolyte on the monolayer is determined via a combination of data for equilibrium spreading pressure and surface pressure versus area isotherms. We show that the monolayer-induced adsorption of electrolyte is not only strongly ion-specific but also surfactant-specific. The comparison between the ion-specific effects on a carboxylic acid monolayer at low pH and an ester monolayer shows that the anion series follows the same order while the cation series reverses. The effect of the electrolyte on the chemical potential of the monolayer shows attraction between the surfactant and the ions at low monolayer densities, but at high surface densities, repulsion seems to come into play. In nearly all investigated cases, a maximum of monolayer-induced electrolyte adsorption is observed at intermediate monolayer densities. This suggests specific interactions between the surfactant headgroup and the ions. The Volta potential data for the monolayers are analyzed on the basis of the equations of quadrupolar electrostatics. The analysis suggests that the ion-specific effect on the Volta potential is due to the ion-specific decrement of the bulk dielectric constant of the electrolyte solution. Moreover, we present evidence that in most cases the effect of the electrolyte on the orientation of the adsorbed dipoles cannot be neglected. Instead, the change in the ion distribution in the electric double layer seems to have a small effect on the Volta potential.

## Introduction

In 1884, Arrhenius presented his dissertation,^1^ where he proposed that salts dissociate into paired ions when dissolved, later published in the first volume of the *Zeitschrift für Physikalische Chemie*.^2^ Building upon his work, the term electrolyte has been introduced as a substance that increases the electrical conductivity of its solution. Electrolyte solutions are a key component of all biological systems, all natural bodies of liquid water contain electrolytes, and aqueous electrolytes are essential ingredients in industrial processes such as flotation, enhanced oil recovery, extraction, and so forth. One important feature of electrolytic solutions, which is still poorly understood, is that their properties vary not only with the ion charge and concentration but also with the ion’s chemical identity, a phenomenon called ion specificity. As far back as 1847, Poiseuille reported that some salts increase the viscosity of water while others decrease it,^3^ which has been cited as the first “scientific observation” of an ion-specific effect.^4^ Since then, ion specificity has been discussed at great length for over 100 years, yet there is no generally accepted first-principles theory of it.

**Figure 1:**
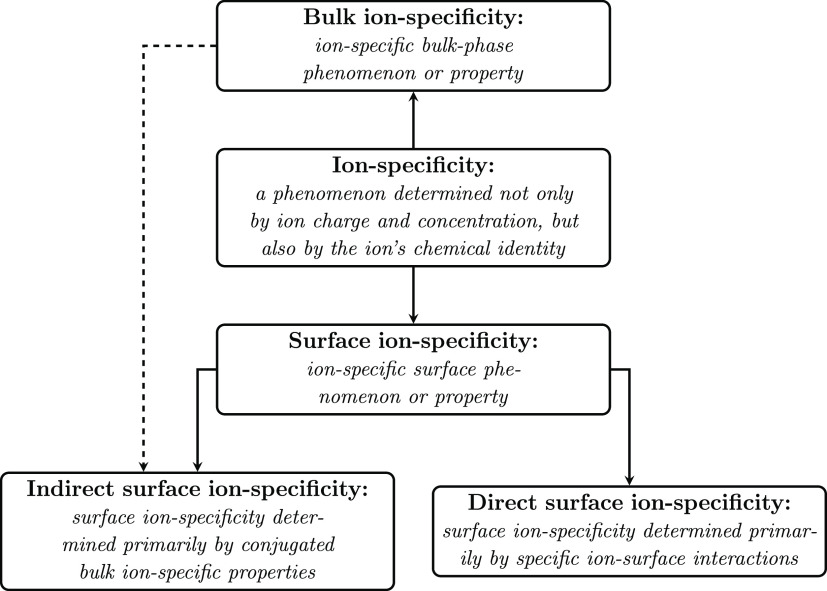
Classification of ion-specific phenomena.

Since the progress in the field seems to be impeded in part by the lack of standardized terminology,^5^ we feel that the terminology we use requires a careful definition. Ion specificity can be divided into bulk ion specificity and surface ion specificity, referring respectively to bulk properties (such as viscosity, dielectric permittivity, activity coefficients, etc.) and surface properties (such as surface tension, adsorption, surface potential, etc.); see Figure 1. When it comes to the ion specificity of surface properties, one would intuitively expect that it is caused by the ion specificity of the underlying surface interactions. However, often this is not the case. For instance, the ion specificity of the surface Δχ potential of simple electrolytic solutions is a consequence of the ion-specific bulk dielectric permittivity, while the ion distributions near the surface and their adsorption play a secondary role.^6^ Thus, we will distinguish between direct and indirect surface ion specificity. If the ion specificity of a surface property is produced by a specific ion–surface interaction, then we speak of direct surface ion specificity. This situation is typical for complex phenomena, e.g., the stability of protein solutions upon addition of salt following the Hofmeister series.^7^ In contrast, we call indirect surface ion specificity one that is controlled by a conjugated bulk property. A natural question arises here: what is the simplest system showing surface ion specificity that can be classified as direct?

It is also useful to classify the ions based on their surface behavior. They can be put on a spectrum from surface-active (large organic ions/ionic surfactants) to surface-inactive (small inorganic ions). Salts composed of the former lead to a significant decrease of the surface tension σ of water, while those of the latter increase it. The surface-inactive monovalent ions are the ones of bare ionic radius <2 Å.^8^ The ions of intermediate size (radius >2 Å; I^–^, ClO_4_^–^, SCN^–^, etc.) form a third group that we will call sticky ions, following Leontidis et al.^9^ Sticky ions have an increased affinity to the interface compared to that of surface-inactive ions. However, their effect on the interfacial tension is small compared to that of surface-active ions, and its direction depends on the nature of the interface and the co-ion. The hydronium ion is an exception to this size-based classification because H_3_O^+^ behaves as sticky despite its small size. All electrolytes that are a combination of surface-inactive ions exhibit surface tension that is indirectly surface-ion-specific and controlled by the bulk electrolyte activity coefficients.^6,8^ On the other hand, the sticky ions adsorb specifically, which can produce a direct surface ion specificity of σ, Δχ, etc.

**Figure 2:**
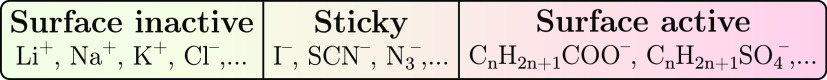
Classification of the ions with respect to their affinity to the W|A surface.

In the past 30 years, a variety of spectroscopic techniques have been employed to elucidate the ion distribution on the water|air (W|A) surface, as reviewed by Petersen et al.^10^ Two that gained much attention are sum frequency generation (SFG) and its variant, second harmonic generation (SHG), which can be classified as purely surface spectroscopies, as signal is detected only from noncentosymmetric media (only on the surface for fluid interfaces). SFG was used to demonstrate that the addition of an electrolyte to the solution augments little to not at all the signal from the free dangling −OH groups at the topmost layer.^11,12^ This has been interpreted as evidence for a greatly diminished presence of ions in the surface region compared to in the bulk, in agreement with surface tension measurements. In a more detailed study, Allen et al. show that with the addition of Br^–^ and I^–^ the size of the probed interfacial region increases.^13^ They interpret the result as these ions having a higher concentration in the probed part of the surface region compared with the bulk. The SHG technique has also been used to study the W|A interface in the presence of electrolytes,^14−17^ seemingly confirming the specific adsorption of sticky anions on W|A.

Hemminger et al.^18^ used X-ray photoelectron spectroscopy (XPS) to study the Br^–^ and I^–^ solutions. Their experiments suggest the specific adsorption of these two ions. Konovalov et al.^19^ probed the W|A surface with grazing-incidence X-ray fluorescence (GIXF). They show that the normalized intensity of the Li^+^, Na^+^, K^+^, and Cs^+^ signals is smaller on the surface than in the bulk. The same is valid for the signals from Cl^–^, Br^–^, and I^–^ (with the exception of HCl). Assuming the absorption coefficients of the ions on the surface and in the bulk are the same, this can be interpreted as a lower concentration of the ions in the surface region. Furthermore, they show that the larger sticky anions (Br^–^ and I^–^) have a higher surface concentration than Cl^–^. The signal strengths suggest that, near the surface, there are more cations than anions. Similar relations hold true for KCl in a critical binary mixture of water and 2,6-dimethylpyridine:^20,21^ the interfacial concentration of ions is diminished near the surface, and close to the surface plane the cation produces higher relative signal than the anion.

Regardless of this diversity of spectroscopic techniques, ion concentration profiles still seem to be experimentally inaccessible. The spatial resolution of these techniques is comparable to or greater than the characteristic length of the ion distributions near interfaces (on the angstrom scale for concentrations above 300 mM), further complicated by the fact that the roughness of liquid interfaces is also of the same order of magnitude due to thermally activated capillary waves.^22^ The result is that the spectroscopic data are difficult to compare to models of the surface of electrolyte solutions. The validation of such models still relies on macroscopic parameters–surface tension and Δχ potential data^6,23−25^–and in that case, the information for the ion–surface interaction potentials and the resulting ion profiles is largely lost (lumped into an integral quantity). Very different model interaction potentials can produce the same integral excess ion concentrations.

Tensiometry appears to be the simplest method able to provide assumption-free quantitative information about the interaction between ions and liquid interfaces. An electrolyte made of surface-inactive ions increases the surface tension σ of water, according to Gibbs’ isotherm
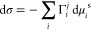
1where  is the surface excess of the concentration of the *i*th species in the solution with respect to the dividing plane *j* and  is the chemical potential of the *i*th species (*i* runs over all species in the system). At chemical equilibrium, the bulk  and surface  chemical potentials are equal, thus we will omit the superscript where not necessary. Since macroscopically the effect of the cations and anions cannot be separated, the corresponding average variables are introduced:  is the surface excess of electrolyte (sum over the ions only) and μ_el_ ≡ *∑*_*k*_ν_*k*_μ_*k*_/ν is the chemical potential of the electrolyte, where ν_*k*_ is the stoichiometric coefficient of the *k*th ion and ν = *∑ν*_*k*_ is the isotonic coefficient. For the adsorption of electrolytes at the equimolecular surface of water, Gibbs’ isotherm becomes
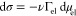
2where Γ_el_ is the surface excess of the electrolyte. (Throughout this article, where no superscript is present, the surface excess is defined with respect to the water equimolecular surface.) The chemical potential of the electrolyte μ_el_ grows with an increase in concentration. Therefore, a rise in the surface tension σ on W|A corresponds to negative Γ_el_; i.e., the electrolyte desorbs and the ions are depleted in the surface region. According to the three-layer model of the surface,^26,27^ the electrolyte adsorption Γ_el_ has contributions from the depletion, the specific adsorption, and the diffuse adsorption layers. The ion-free depletion layer is the result of hydration and image forces acting on the ions. The specific adsorption layer is the result of short-range specific interactions of the ions with the surface, as demonstrated by molecular dynamics simulations for halogens.^24,28,29^ Finally, the diffuse layer is produced by the uncompensated for charge of the depletion and adsorption layers. The negative value of Γ_el_ does not necessarily mean that no ions are specifically adsorbed in the adsorption layer; for example, H_2_SO_4_ has overall negative adsorption^30^ due to the depletion of  winning over the positive specific adsorption of H_3_O^+^.

The chemical potential of the electrolyte is related to its bulk concentration *C*_el_ through
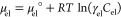
3Here, μ_el_° is the standard chemical potential of the electrolyte, *R* is the universal gas constant, *T* is the temperature, and γ_el_ is the bulk mean activity coefficient of the electrolyte. Combining eqs 2 and 3, the surface excess of an electrolyte Γ_el_ can be determined from experimental data for σ(*C*_el_) and γ_el_(*C*_el_). In the literature, this is often done by assuming unity for the activity coefficients. Thus, the ion specificity of the slope of σ(*C*_el_) should correspond to the ion specificity of Γ_el_. However, the approximation γ_el_ = 1 fails at *C*_el_ > 300 mM and even earlier for multivalent salts. Using experimentally measured activities, it was shown that the ion-specific ordering of σ(*C*_el_) for surface-inactive ions directly corresponds to that of γ_el_(*C*_el_);^6,8^ i.e., the surface ion specificity of σ is indirect, as it is produced by the specificity of the bulk electrolyte activities.

**Figure 3:**
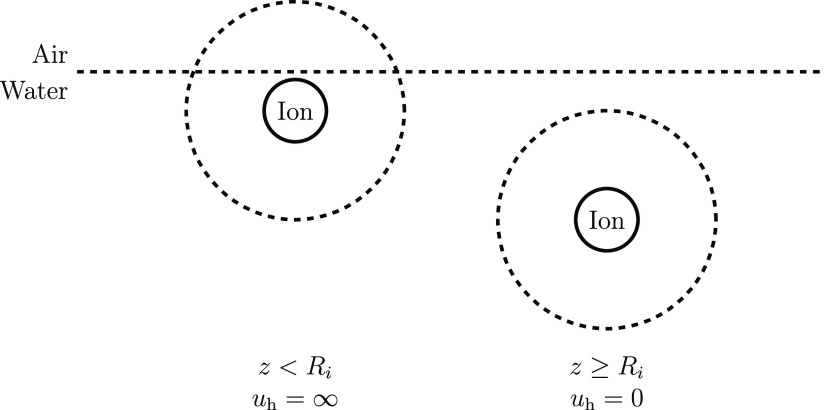
Schematic of the repulsive hydration potential acting on an ion near the W|A interface. Left: at position *z* < *R*_*i*_, the ion has an incomplete hydration shell and infinite hydration potential. Right: at separations from the surface larger than *R*_*i*_, the ion has a complete hydration shell and is effectively a bulk ion.

In general, the limiting models of electrolytic solutions are based on point charge interactions in continuous media and predict rigorous relations for the solution properties at low concentrations, where ion specificity is absent. When it comes to electrolyte adsorption and surface tension, the limiting model is that of Onsager and Samaras.^31^ The interface is presented as a mathematical plane between two media of different dielectric permittivities. The ions are subject only to the longest-ranged forces—image forces (i.e., the ion charge/solvent dipole interactions). The net force acting on the ion is directed toward the more polarizable medium (water). Numerous extensions to the Onsager–Samaras model have been proposed to accommodate ion specificity. Most of them focus on additional ion-specific interactions: dispersion,^23,32−38^ hydrophobic,^38^ static polarization,^25,38^ etc. Unfortunately, all of these models seem to be parametrized, often with several free parameters. Since σ(*C*_el_) is approximately linear, a single sensitive regression parameter is sufficient to describe this dependence. This makes the discussion about which ion-specific interactions are “important” speculative. The simple Onsager–Samaras model can be improved significantly by allowing for the discrete structure of the ion hydration shell: when an ion approaches the interface, its energy rises in a stepwise fashion, corresponding to removing a whole number of water molecules from the hydration shell. This we designate as the hydration potential. In the simplest approximation, it is a potential profile with a single step from null to infinity, producing a perfect ion-free depletion layer (Figure 3). Using this hard-wall potential, in 1955, Schmutzer^39^ improved upon Onsager–Samaras’ model and showed that the modified version appears to have a significantly wider range of validity and allows for ion specificity. However, Schmutzer treated the size of the depletion layer as a free parameter. Recently, a direct relationship among the size of the depletion layer, the bare ion radii, and the structure of the water surface has been proposed.^6,8^ The result is a quantitatively predictive model for the ion-specific desorption of surface-inactive ions at W|A with no free parameters.

Simple uncharged monolayers on W|A (designated as W|M) are prime candidates for a system where a direct surface ion specificity might be present even for surface-inactive ions. Surface XPS experiments have suggested specific interactions between ions and butanol.^40^ Furthermore, the nature of the organic surfactant may result in diametrically opposite interactions with the ions: butanol causes a surface enhancement of the Br^–^ and I^–^ signals, and butanoic acid causes a surface diminution.^41^ There have been a handful of tensiometric studies demonstrating the ion-specific effects on W|M for uncharged monolayers. In 1939, Pankratov and Frumkin observed that the surface pressure π(Γ_s_, *C*_el_) = σ(0, *C*_el_) – σ(Γ_s_, *C*_el_) at a constant area of the monolayer *S* (*S* = 1/Γ_s_, where Γ_s_ is excess surfactant) increases with the addition of electrolyte in the substrate.^42,43^ Later, Donnison and Heymann presented a systematic study of the effect of electrolyte on the equilibrium spreading pressure of surfactants.^44^ They reported a linear increase in the spreading pressure with the concentration of the electrolyte. The slope correlates well with the energy of hydration; more polarizable ions with a lower energy of hydration (e.g., Rb^+^ and SCN^–^) increase the spreading pressure more. However, this ordering is not general but depends on the surfactant. Later, Gilby and Heymann studied more electrolytes (including polyvalent) and measured complete surface pressure to area isotherms for oleic acid.^45^ Their results show that the area of collapse of the monolayer is hardly changed by the electrolyte, suggesting that the ions do not change the structure of the films near collapse. The effect of the electrolyte is greater on the more dilute monolayer. The same finding was reported for lipid monolayers.^46−48^ Raltson and Healy investigated the effect of cations on octadecanol monolayers.^49^ They report a correlation between the electrolyte effect on the monolayer and its effect on the W|A surface tension. Peshkova et al.^27^ revisit and extend Raltson and Healy’s results to find that the electrolyte surface excess changes with the density of the monolayer in a nonmonotonous manner. The authors argued that such behavior cannot be explained with the four major forces assumed to control the electrolyte behavior at W|A (image, hydration, hydrophobic, and dispersion interactions) and concluded that there must be another, previously unstudied significant interaction between monolayers and ions. More recent studies on this type of system focus on zwitterionic phospholipid monolayers due to their biological relevance.^46−48,50−55^ However, those are complicated by the possibility of strong ion–surfactant coordination and partial protonation of the phosphate group. They are left as a separate future prospect, while here we focus on simpler nonionic insoluble surfactants.

The picture that is emerging is of specific interactions between the ions and surfactant moieties that result in direct ion specificity of the surface tension even for surface-inactive electrolytes. To study these interactions in a top-down approach, a well-developed methodology of calculating the electrolyte surface excess from tensiometric data is needed. To that end, we further expand and elaborate on the only available method for this calculation.^27,42^ Using this methodology, we present an analysis of available literature data for surface pressure to area isotherms, equilibrium spreading pressure, and Δ*V* potential for different uncharged surfactants in the presence of electrolytes. (refer to Table S1 for a compilation of the experimental data sources). We demonstrate that ions that show no specific interaction with W|A and water|oil (W|O) interfaces interact highly specifically with monolayers. The presentation starts with a general thermodynamic consideration of the W|M system as well as the theoretical basis for the numerical procedure for calculating the excess electrolyte on the monolayer. The results and their discussion are divided into three parts: equilibrium spreading pressures, the surfactant surface pressure to area isotherms, and the Δ*V* potentials.

## Methods

Recently, we revisited Schmutzer’s model^39^ and improved upon it by further specifying the depletion and diffuse ion layers.^6,8^ First, the thickness of the depletion layer (an average value in Schmutzer’s model) was explicitly related to the thicknesses *R*_*i*_ of the depletion layers for the anion and the cation. The depletion thicknesses *R*_*i*_ were calculated from the size and charge of the ions. The radial distribution function of water around an ion has one strong peak for monovalent ions^56^ and two strong peaks for divalent ions.^57^ Thus, the assumption made is that monovalent ions can shed all but the last hydration shell and that multivalent ions retain two complete shells when approaching the interface. The size of the hydrated ion *R*_h,*i*_ can be found from geometric considerations^6,8^ as
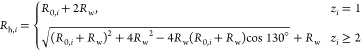
4where *z*_*i*_ is the charge of the ion (by absolute value), *R*_0,*i*_ is the crystallographic ionic radius of the *i*th ion, and *R*_w_ is the effective radius of water, 1.39 Å.

Within Schmutzer’s model, the location of the hard wall of the hydration potential corresponds to the plane of discontinuity of the dielectric permittivity ϵ. At hydrophobic|aqueous interfaces, there is a surface deficit of water density, known as a hydrophobic gap. The size of the hydrophobic gap has been shown experimentally to correspond approximately to the effective radius of a water molecule.^58^ Therefore, the plane of discontinuity of ϵ is placed in the middle of the last layer of the water molecules. The thickness of the depletion layer *R*_*i*_ is the distance from the plane of discontinuity of ϵ to the position of the closest approach of the *i*th ion, which is *R*_h,*i*_ away from the top of the last layer of water molecules. This leads to
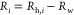
5This modified Schmutzer (MS) model predicts the surface tension of surface-inactive electrolyte solutions with no adjustable parameters. It has been verified against experimental results for many electrolytes and has been shown to predict quantitatively the surface tension for any combination of Li^+^, Na^+^, K^+^, Rb^+^, Cs^+^, Mg^2+^, Ca^2+^, Ba^2+^, La^3+^, OH^–^, F^–^, Cl^–^, Br^–^, NO_3_^–^, and CO_3_^2–^ ions up to 1–5 mol/kg. The model is valid for all monovalent ions of bare radius *R*_0,*i*_ < 2 Å, justifying the boundary *R*_0,*i*_ = 2 Å between surface-inactive and sticky ions. Furthermore, the model was found to work similarly well at the W|O interfaces.^59^

Within the MS model, the surface excess of electrolyte , with respect to the ϵ plane of discontinuity, is calculated as
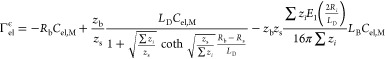
6where *R*_*i*_ and *z*_*i*_ are the depletion layer thickness and the charge of the *i*th ion, respectively. Subscripts b and s designate the bigger and smaller ions, respectively. *E*_1_ is an exponential integral of the first order. *L*_B_ ≡ *e*^2^/*ϵkT*, where *e* is the elementary charge, is the Bjerrum length.  is the Debye length of the solution, where *N*_A_ is Avogadro’s number and  is the ionic strength in mol/m^3^. Subscripts *m* and M differentiate between molal and molar quantities, respectively. Furthermore, the surface excess Γ_el_ at the equimolecular surface is related to  through^8^
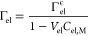
7where *V*_el_ is the bulk partial molar volume of the electrolyte. The correction factor 1 – *V*_el_*C*_el,M_ is due to the shift of water’s equimolecular plane toward the solution upon increasing the electrolyte concentration. In eq 6, one can find the corresponding electric double layer surface potential  as
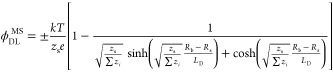
8where the sign is determined by the polarity of the smaller ion (if the cation is smaller, the formula starts with a plus sign).^6^

The hydration force is of the same nature (ion/dipole interaction) as the image force but over a different range. Within the MS model, the range of the hydration potential is derived on the basis of considerations about the structure of the interface and the ion hydration shell. Thus, the hydration force is directly surface-ion-specific but does not vary much from ion to ion. As a result, the ion specificity of the surface tension increment is determined mostly by the bulk activity of the ions.

Within the framework of the MS model, the addition of an uncharged monolayer should have a small effect on the two underlying interactions.^59^ The image force is controlled by the difference in bulk dielectric permittivities of water and the hydrophobic phase and does not change in size or direction if a monolayer is present. However, the monolayer alters the profiles of ϵ and of water density in the vicinity of the surface, shifting the position of the water equimolecular surface with respect to the ϵ plane of discontinuity. In order to relate the electrolyte adsorption at these two planes, we use Gibbs’ isotherm (eq 1) for each plane
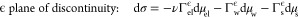
9
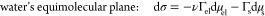
10where Γ_s_ is the surface excess of the surfactant with respect to water’s equimolecular surface,  is the surface excess of surfactant with respect to the plane of ϵ discontinuity,  is the surface excess of water with respect to the plane of ϵ discontinuity, μ_s_ is the chemical potential of the surfactant, and μ_w_ is the chemical potential of water. For an insoluble surfactant, eqs 9 and 10 can be rearranged to
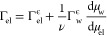
11since . Furthermore, the bulk Gibbs–Duhem relation reads
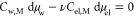
12where *C*_w,M_ is the bulk concentration of water. Combining eqs 11 and 12, we obtain
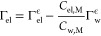
13The following general relation should hold for all *z*
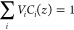
14where *V*_*i*_ is the partial molar volume of the *i*th component. Assuming the partial molar volumes *V*_*i*_ are constant, we can integrate eq 14 with respect to *z* up to the ϵ discontinuity surface to obtain (section S3.3)
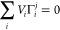
15By combining the last result with eqs 13 and 14, we arrive at
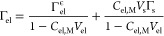
16Here, *V*_s_ is the partial molar volume of the polar headgroup (the volume of the surfactant that remains below the surface of ϵ, see section S3.3). The second term can be viewed as an osmotic effect on Γ_el_ from the surfactant headgroups, diluting the solvent in the surface layer.

When it comes to the hydration force, the effect of the monolayer is more complicated. It seems reasonable to assume that the polar headgroups of the surfactant can be incorporated into the solvation shell of the ions. Therefore, the solvated radius *R*_h,*i*_ (eq 4) will change, and so will the hydrophobic gap thickness, both altering *R*_*i*_. Moreover, the difference in the solvation energy on the surface (in the presence of surfactant) and in the bulk will produce an ion-surface solvation potential, *u*_s_. This solvation interaction may increase or decrease specifically the ion adsorption, depending on whether the ions are solvated better on the surface or in the bulk. For instance, on the W|A surface with adsorbed hexanol, MD simulations show a decrease in the average coordination number of the ions,^60^ suggesting a net repulsion from the surface.

Literature data for insoluble monolayers are reported in terms of the surface pressure π. Equation 10 can be written as
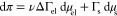
17where  is what we call the monolayer-induced adsorption of the electrolyte (with respect to the equimolecular surface). ΔΓ_el_ is the extra electrolyte attracted to the surface when a monolayer is spread on W|A. It is an indicator of the interactions between the ions and the monolayer. However, it should be kept in mind that the position of the water equimolecular dividing plane is not the same with respect to the plane of ϵ discontinuity for W|M and W|A.

For the W|M system, interpreting the tensiometric data thermodynamically is more intricate than that for the simple W|A system. In a two-component system, the electrolyte adsorption is fully defined by σ(μ_el_). When a third component (the surfactant) is present, the data for σ(*C*_el_) are no longer sufficient to deduce Γ_el_ unless the chemical potential of the third component is constant with *C*_el_. From eq 17, it follows that
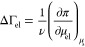
18The differential with respect to μ_el_ can be converted to a more practical differential with respect to the molality *C*_el,*m*_ or osmotic pressure *p*_osm_. Thus, using the relation dμ_el_ = d*p*_osm_/ρ_w_*νC*_el,*m*_ in eq 18, the monolayer-induced adsorption of electrolyte can be calculated as
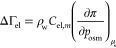
19where ρ_w_ is the mass density of water. The osmotic pressure is defined as^61^
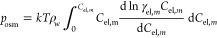
20Unfortunately, the chemical potential of the surfactant μ_s_ can be kept constant experimentally only in special cases. For W|A, one way to achieve this is by dispersing on the surface a powder or droplets of the surfactant phase. The surfactant then spreads on the free surface to form the so-called equilibrium spread monolayer with a spreading pressure π_sp_. Then, eq 19 can be used to calculate the monolayer-induced adsorption of the electrolyte. However, when working with equilibrium spread monolayers, the monolayer density is not a controllable parameter. In fact, the equilibrium spread monolayer is in general close to the collapse point of the monolayer (i.e., densely packed).

The interaction of ions with less dense spread monolayers can be studied via the surface pressure to area isotherms of the surfactant at different salt concentrations. However, extracting the excess electrolyte from them is not trivial. From eq 17, one can derive two more useful partial differential relations:
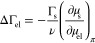
21
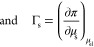
22Pankratov and Frumkin proposed a method of extracting the surfactant chemical potentials by combining the surface pressure to area isotherms with data for the equilibrium spreading pressure,^43^ based on eq 22 in the form
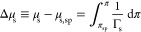
23Here, μ_s,sp_ is the chemical potential of reference state π_sp_. Once Δμ_s_ is known, one can use eq 19 to calculate ΔΓ_el_. Relation 21 provides a second way to determine the monolayer-induced adsorption of the electrolyte:
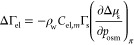
24In order to use eqs 23, 19, and 24 to determine ΔΓ_el_, reference state μ_s,sp_ must be independent of the concentration of electrolyte *C*_el_. The equilibrium spread monolayer is one such state since the electrolyte should not affect the bulk surfactant phase and μ_s,sp_ can be assumed to be constant.

**Figure 4:**
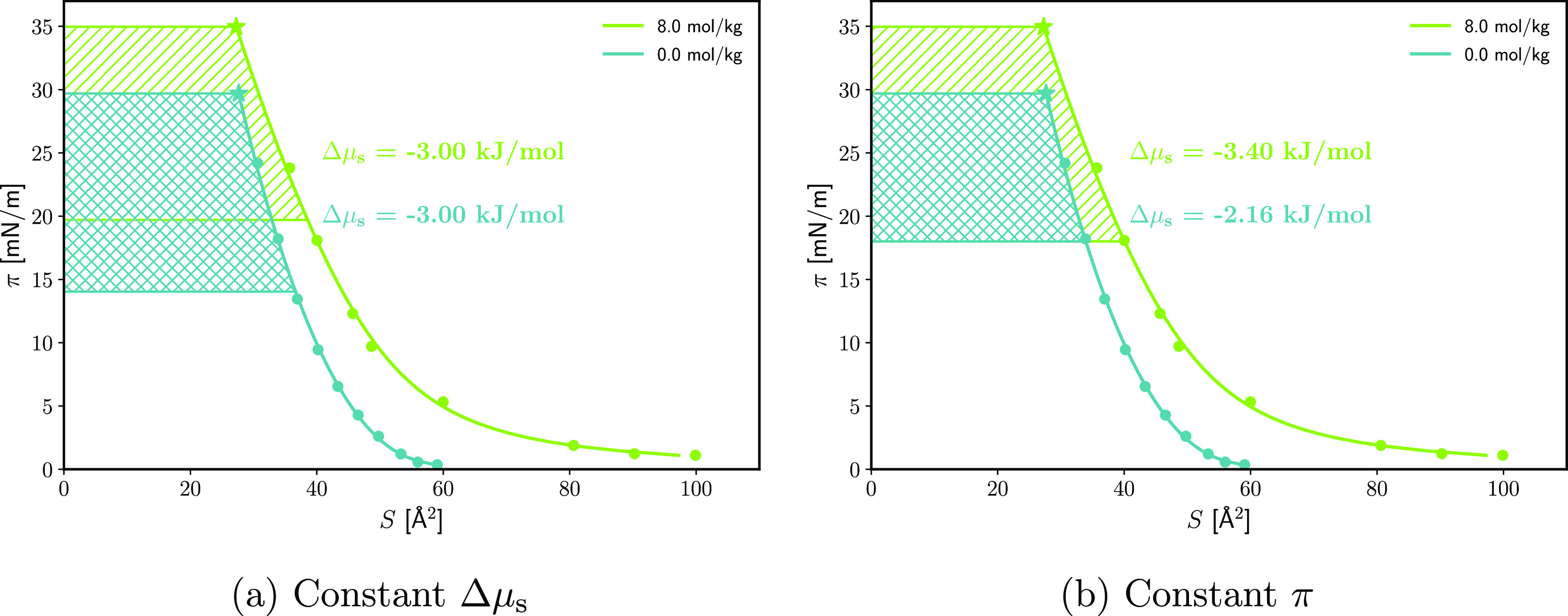
Surface pressure to molecular area isotherms of oleic acid on water and on 8 mol/kg LiCl up to the equilibrium spreading pressure (data from Gilby and Heymann^45^). (a) Demonstration that the surface pressure, for a fixed surfactant chemical potential, increases with the addition of electrolyte (from 14 to 20 mN/m in the example). This allows the computation of Γ_el_ via eq 19. (b) Chemical potential of the surfactant decreases at a fixed surface pressure. This allows the computation of Γ_el_ via eq 24.

In the literature, it is common to compare the variation of the π(Γ_s_) isotherms with *C*_el_ at a constant pressure or constant area. Then, in the absence of direct values for Γ_el_, the effect of the electrolyte on the monolayer is discussed qualitatively, e.g., in terms of a “change in cohesion” of the monolayer. However, in eq 10, the electrolyte alters the surface pressure directly through the νΓ_el_dμ_el_ term as well as indirectly through Γ_s_dμ_s_; i.e., it both translates the isotherm and changes its shape, which complicates such an interpretation. We instead determine the surface excess of electrolyte on the monolayer as follows from eqs 19 and 24. The two approaches are illustrated in Figure 4. The values of ΔΓ_el_ follow the effect of *C*_el_ on the area bound by the isotherm and the π axis. Using eq 19, one compares the surface pressures π that give the same area (Δμ_s_) at different electrolyte concentrations, as proposed by Pankratov and Frumkin.^43^Equation 24 provides another, previously unexplored, route to computing ΔΓ_el_ by comparing the areas (Δμ_s_) at constant surface pressures π and different electrolyte concentrations. Ideally, the two routes should produce the same ΔΓ_el_, but due to the experimental uncertainties, deviation is inevitable. This deviation is a measure of the uncertainty of ΔΓ_el_ and a method to test the thermodynamic compatibility of the two sets of experimental data: π_sp_(*C*_el,M_) and π(*S*).

## Results and Discussion

### Analysis of Equilibrium Spreading Pressure Data

In this section, we calculate ΔΓ_el_ from π_sp_ data using eq 19. The equilibrium spreading pressures for oleic acid (OA) and diethyl sebacate (ES) on various electrolyte solutions were measured by Heymann et al.^44,45^ (20 ± 2 °C, atmospheric pressure). The data for OA are for the subphase acidified with 0.01 M HCl to suppress the dissociation of the organic acid. Since the concentration of HCl is much lower than the electrolyte concentrations, the surface activity of the inorganic acid and its effect on the electrolyte bulk activity are assumed to be negligible (discussion in section S3.1). However, Heymann et al. studied several electrolytes (KSCN, Na_2_SO_4_, and MgSO_4_) that hydrolyze in the presence of HCl: these we exclude from consideration since at high concentration they raise the pH by several units and lead to dissociation of the OA and charging of the monolayer, an effect beyond the scope of this work. The electrolyte activity coefficients necessary in eq 19 were collected from multiple sources (Table S1 in the SI).

**Figure 5:**
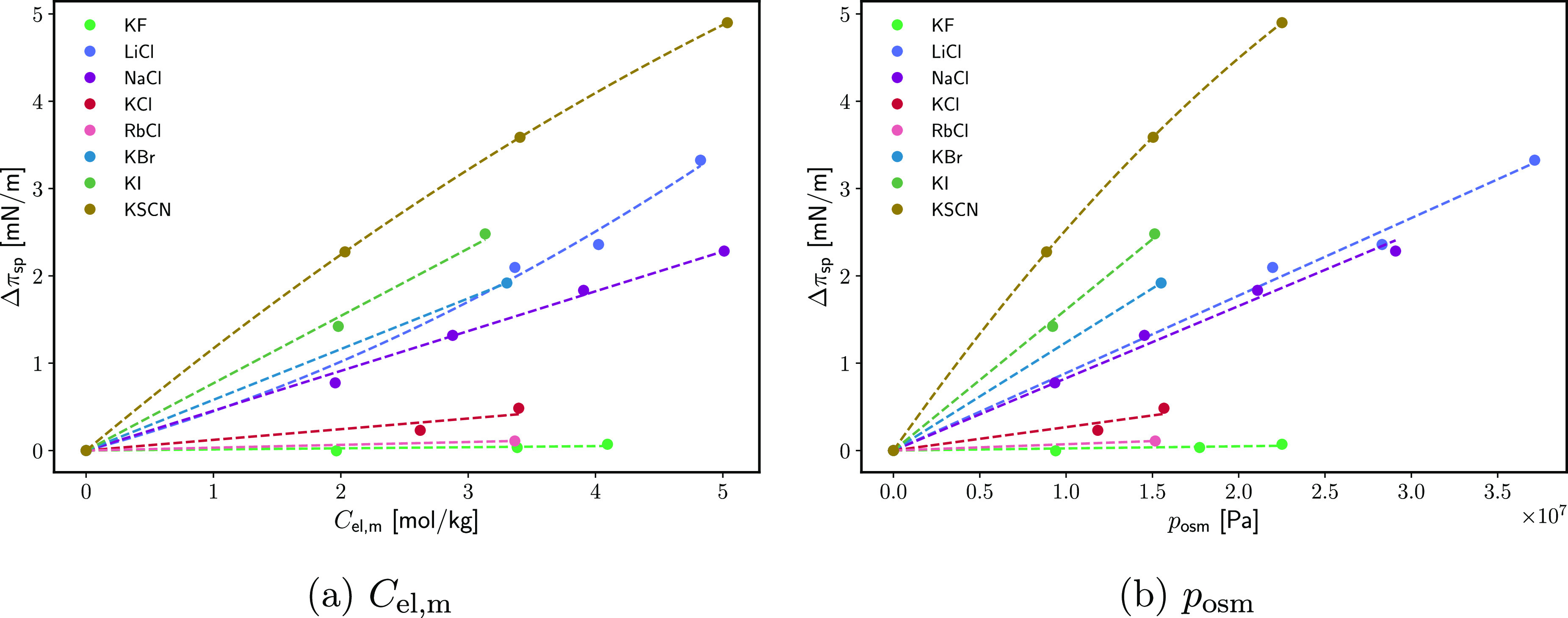
Increase in the equilibrium spreading pressure of ES due to different electrolyte solutions. A side-by-side comparison of (a) Δπ_sp_(*C*_el,m_) and (b) Δπ_sp_(*p*_osm_). The data are from Donnison and Heymann.^44^ The solution concentrations and osmotic pressures are calculated from the activities in ref (44) using literature activity coefficients.

Figure 5 shows the typical effect of the electrolyte on the equilibrium spreading pressure of ES in terms of the increment in Δπ_sp_. What can be seen is that, upon the addition of electrolyte, π_sp_ rises in an ion-specific way. The concentration and osmotic pressure of the solution are calculated from the activity values reported by Heymann et al. using literature activity coefficients (Table S1). The osmotic pressure *p*_osm_ was found to best linearize the data (compare Figure 5 with Figure 2 from ref (44), where *a*_el,m_ is used instead). This finding can be rationalized by the fact that, from eq 19, the slopes in Figure 5 are proportional to the surface excess over molal concentration ∂Δπ_sp_/*∂p*_osm_ ∝ ΔΓ_el_/*C*_el,*m*_ and at high concentration ΔΓ_el_ ∝ *C*_el,M_ (refer to refs (6) and (8)). Therefore, throughout the rest of this article, *p*_osm_ is used as the independent variable for the calculations. The relative effect of the ions on ∂Δπ_sp_/*∂p*_osm_ follows the same ordering as that on *∂π*_sp_/*∂a*_el,*m*_ as reported by Heymann and Donnison, with the exception of the Li^+^/Na^+^ pair:
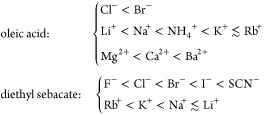


Heymann et al. conclude that the ion-specific effect on π_sp_ follows the “decreasing energy of hydration” with the exception of the cations on the ES monolayer which exhibit “irregular behavior”. However, in π_sp_(*p*_osm_) coordinates the data points for LiCl are above those of NaCl (albeit within the experimental error). Thus, what Heymann et al. call irregular behavior we interpret as a full inversion of the cation series: on the acid, the bigger cations increase the equilibrium spreading pressure more while the opposite is true on the ester. On the other hand, the anion series follows the same order for both amphiphiles. The bigger anions cause a bigger increase in the equilibrium spreading pressure. In contrast, for alcohol monolayers, the anion series is reversed,^27^ with Cl^–^ attracted more to the spread alcohol than Br^–^. This difference between alcohol and acid monolayers is in line with recent spectroscopic studies where the anion “series” was found to invert.^41^

**Figure 6:**
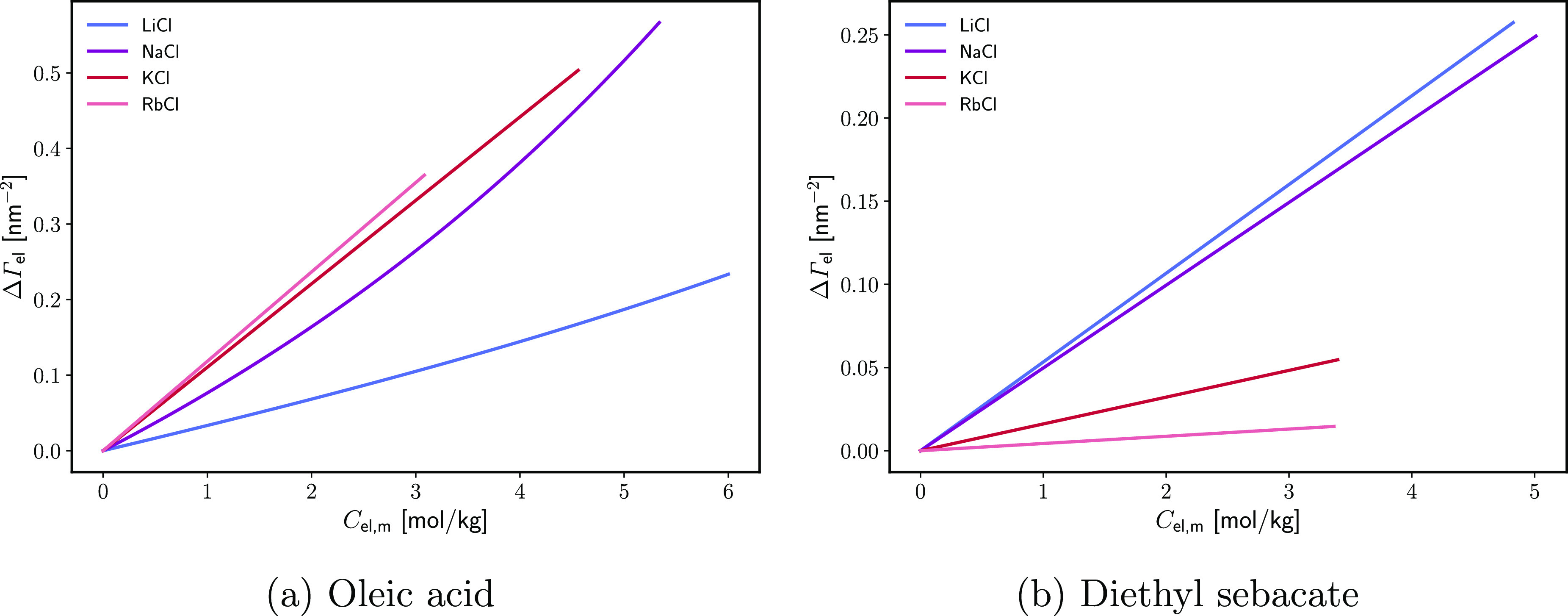
Monolayer-induced electrolyte adsorption ΔΓ_el_ as a function of the electrolyte concentration *C*_el,*m*_ for different monovalent chloride salts. ΔΓ_el_ is calculated using eq 19 from the data of refs (44) and (45).

The results are confirmed by the monolayer-induced adsorptions of electrolyte ΔΓ_el_, calculated using formula 19. Figure 6 compares ΔΓ_el_ as a function of *C*_el,*m*_ for various chlorides on both surfactants. Let us reiterate that this is excess adsorption in comparison with the one at W|A (where eqs 6 and 7 are followed for the inactive electrolytes in Figure 6). Not only is the cation series reversed but the monolayer-induced adsorption ΔΓ_el_ on OA is significantly larger than that on ES (with the exception of LiCl). The increase in ∂ΔΓ_el_/*∂C*_el,*m*_ follows the same series as the increase in ∂Δπ_sp_/*∂p*_osm_. The observed monolayer specificity of the cation series points to direct specific ion/monolayer interactions. The relative effect of the electrolyte on the spreading pressure, for every choice of independent variable, follows the same series as the monolayer-induced electrolyte adsorptions (compare Figures 5 and 6). Therefore, π_sp_ is a directly surface ion-specific property even for the smallest surface-inactive ions; this is not the case at surfactant-free W|A or W|O.^6,59^

The monolayer-induced adsorption of electrolyte ΔΓ_el_ is a convenient property to calculate and compare between electrolytes but does not provide the direction of the ion/interface interactions on its own. The reason is that the presence of a surfactant changes the position of the water equimolecular plane with respect to the position of the surface of the ϵ discontinuity. Therefore, ΔΓ_el_ compares two surface excesses defined with respect to dividing planes at different distances from the dielectric surface. Thus, ΔΓ_el_ > 0 on its own does not necessarily translate to attraction between the monolayer and the ions. The direction of the effective ion/monolayer interaction can be determined by the sign of the monolayer-induced adsorption  with respect to the plane of ϵ discontinuity. One can relate  to ΔΓ_el_ from eq 16 in the presence of a monolayer:
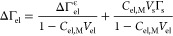
25The term 1 – *C*_el,M_*V*_el_ quantifies the effect from the shift of the water equimolecular plane with the addition of electrolyte.^6,8^ The partial molar volume of the electrolyte can be both positive and negative, thus raising or lowering ΔΓ_el_ in comparison to . The second term in the equation is the result of the shift of the equimolecular surface toward the electrolyte solution due to the expulsion of surface water by the surfactant polar group. This effect is actually a dominant contribution to ΔΓ_el_.

The partial molar volume *V*_s_ and the surface concentration Γ_s_ of the surfactant are needed to evaluate the relative excess with respect to the ϵ plane of discontinuity. The latter could be extracted from the surface pressure versus area isotherms. The collapse area of OA is 27.6 Å^2^, approximately independent of the electrolyte.^45^ The partial molar volume *V*_s_ of the surfactant headgroup is more difficult to determine. Data for partial molar volumes for pure substances and molecular segments are readily available,^62,63^ but it is unclear what fraction of the polar group remains immersed below the surface of ϵ. Considering the carboxylic group of OA, the very least one can imagine entering into the polar phase is the −OH moiety. In that case, the molar volume is approximately equal to the molar volume of water (18 mL/mol). In the other limiting case, the entire −COOH moiety belonging to the polar phase corresponds to the upper bound of *V*_s_, equal to the partial molar volume of formic acid HCOOH in water (35 mL/mol^63^). The actual value of *V*_s_ can be expected to lie between these two limits depending on where the plane of ε discontinuity lies with respect to the α carbon. A rough approximation can be made on the basis of a dielectric multilayer model that considers the headgroups and the hydrocarbon tails as thin layers of specific dielectric permittivity, lower than that of water (described in section S3.3); it produces *V*_s_ = 32 mL/mol.

**Figure 7:**
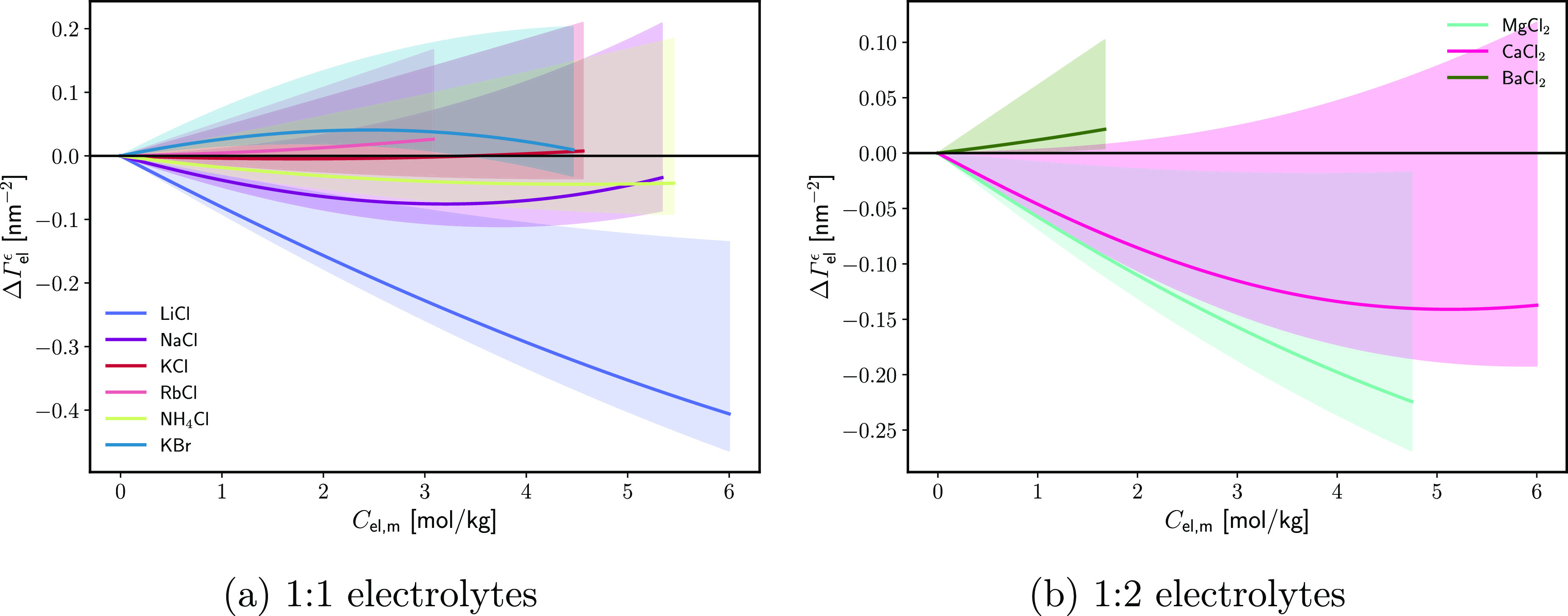
Increase in electrolyte surface excess with respect to the ϵ plane of discontinuity on OA monolayers compared to W|A  as a function of the electrolyte concentration *C*_el,*m*_. Data from refs (44) and (45). The solid lines are calculated using eq 25 with a 32 mL/mol partial molar volume of the surfactant. The area between the two limiting values for the partial molar volume are shaded semitransparently.

Figure 7 compares the monolayer-induced adsorption of electrolyte on OA with respect to the plane of ϵ discontinuity . The semitransparent areas represent the excesses bound by the two limiting molar volumes of the surfactant headgroup, 18 and 35 mL/mol. The solid lines are calculated with the rough estimate *V*_s_ = 32 mL/mol. The values of  at the dielectric plane follow the same order as that of ∂ΔΓ_el_/*∂C*_el,*m*_ with respect to the electrolyte identity. Unlike ΔΓ_el_,  is not strictly positive, a conclusion valid irrespective of the choice of the value of *V*_s_ within the set boundaries. For chlorides of small alkali and alkaline earth metals (LiCl, MgCl_2_, and CaCl_2_) it is negative . Therefore, they have a higher affinity to the W|A interface than to the W|OA interface. The chlorides of larger metals (RbCl, BaCl_2_) have a higher affinity to the W|OA interface. In between, the near-zero slope  for KCl could be explained in two ways: (i) neither K^+^ nor Cl^–^ ions exhibit a preferential affinity to either type of interface or (ii) the adsorption of one ion negates the desorption of the other, e.g., the K^+^ ion is attracted to the monolayer just as much as the Cl^–^ is repulsed by it. Such behavior suggests an interplay between a repulsive and an attractive interaction between the ions and the monolayer. At least one of those interactions must be ion-specific. Furthermore, the linear initial slope  suggests that the ion/monolayer interactions are concentration-independent, up to 2 M (section S3.2).

### Analysis of Data for Surface Pressure π vs Area *S* Isotherms

In this section, we calculate ΔΓ_el_ as a function of the monolayer density Γ_s_ from the pressure to area π(*S*, *C*_el,*m*_) isotherms using eqs 19 and 24. The OA isotherms are from Heymann et al.;^45^ the ethyl palmitate (EP) isotherms are from Frumkin and Pankratov.^42,43^ All experiments were performed at 20 ± 2 °C. The OA data are for the subphase acidified with 0.01 M HCl to prevent dissociation of the OA monolayer. The effect of HCl is again assumed to be negligible (section S3.1). We consider only electrolytes for which two or more concentrations are sampled, as the calculation of ΔΓ_el_ requires numerical differentiation with respect to the concentration of electrolyte ( eqs 19 and 24). To evaluate ΔΓ_el_, the π(*S*, *C*_el,*m*_) data are supplemented with π_sp_(*C*_el,*m*_) data from the same sources. In the case of EP, Frumkin and Pankratov reported only π_sp_ at the highest electrolyte concentration that they studied and for neat water. The intermediary concentrations are interpolated assuming a linear dependence of π_sp_ on *p*_osm_ (Figure 5).

**Figure 8:**
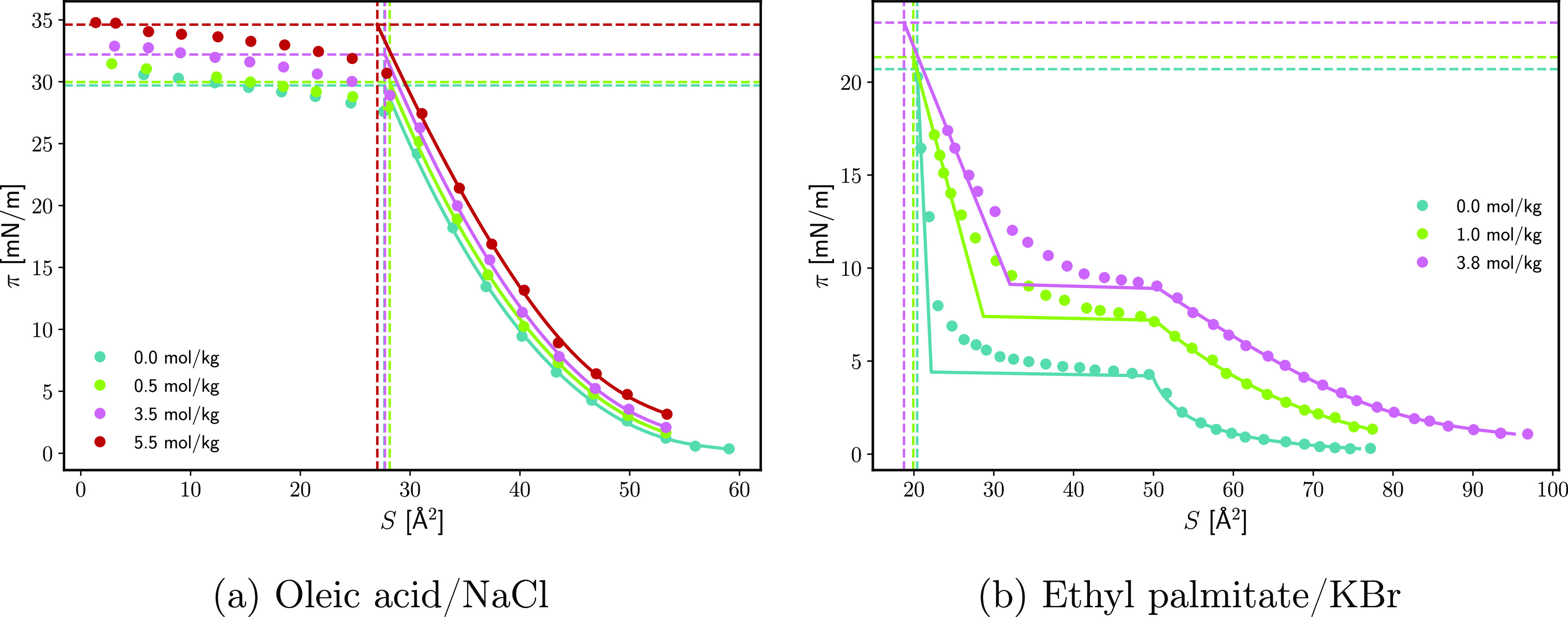
Surface pressure of the M|W interface π as a function of the molecular area of the surfactant *S* at different concentrations of the electrolyte in the subphase. The points are data from refs (42) and (45). The dashed curves are an interpolation of the data. The dashed horizontal lines are the interpolated and measured spreading pressures.^42,45^ The dashed vertical lines are the extrapolated spreading areas.

Figure 8 shows selected π(*S*, *C*_el,*m*_) isotherms. The addition of electrolyte shifts the isotherms upward, which appears to be a general behavior found also for alcohols^27^ and phospholipids.^48,52,53^ At the studied temperature, the EP monolayer goes through a phase transition from liquid expanded to 2D solid, while OA does not. As usual, the phase-transition plateau of the experimental isotherms deviates from being flat, which is assumed to be a kinetic 2D capillary effect related to the high stability of the 2D solid-in-2D liquid dispersion.^27,64^ The isotherms are corrected for this effect (refer to corresponding section S2.5). The dashed lines in the plots indicate the state of the equilibrium spread monolayer. For OA, a collapse is observed just below π_sp_.^45^ The EP data are reported before collapse and below the equilibrium spread pressure.^42^

**Figure 9:**
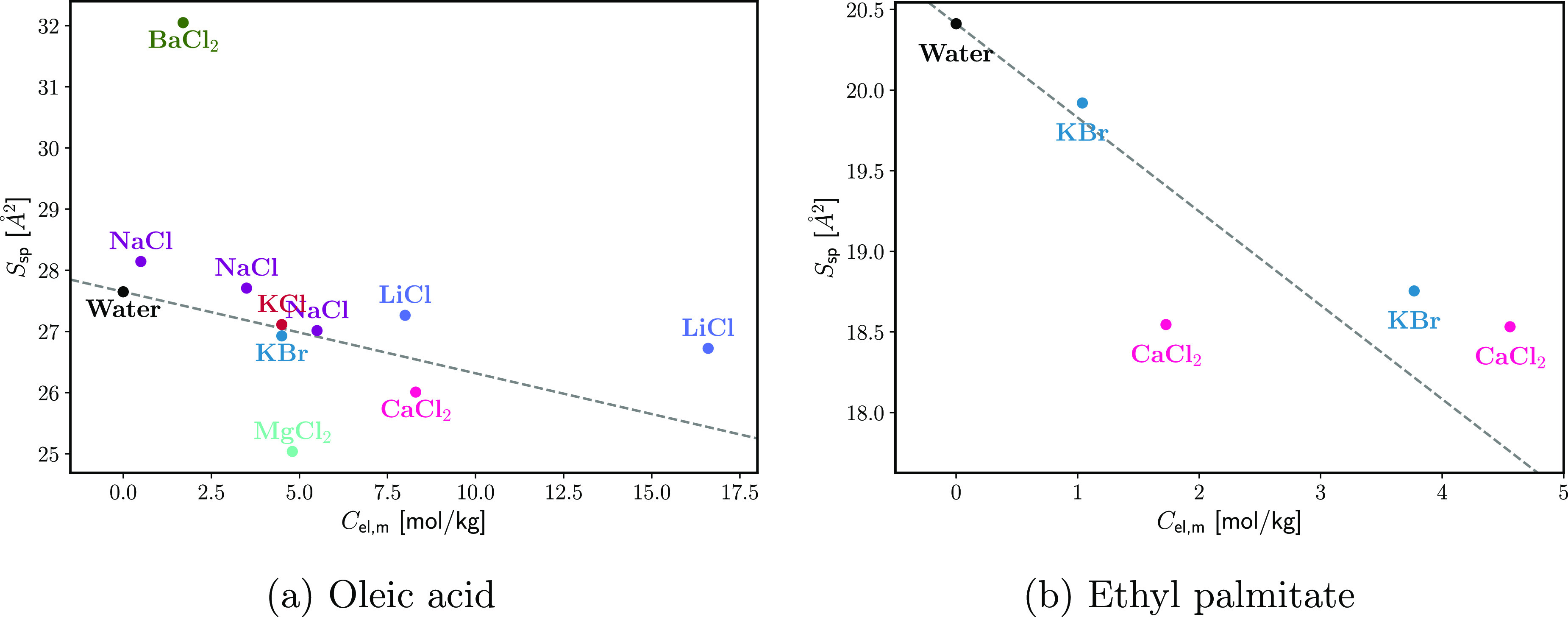
Area per molecule in an equilibrium spread monolayer *S*_sp_ (in equilibrium with bulk surfactant phase) as a function of the electrolyte concentration *C*_el,*m*_. Data are from refs (42) and (45).

The areas per molecule *S*_sp_ that correspond to the equilibrium spreading pressures were found by extrapolating to the intersection point with the horizontal π_sp_ lines (Figure 8). This is done by assuming a linear π(*S*) relationship for the elastic 2D solid phase close to the collapse/crystallization point: a line is drawn through the last two to three points before the collapse and extrapolated to the spreading pressure. Figure 9 shows the so-obtained values of *S*_sp_ for all electrolytes in the source data. *S*_sp_ was found to decrease with the addition of electrolyte but by no more than 10% from the neat water value. The only notable exception is BaCl_2_ on OA, where *S*_sp_ increases by about 15%. In this case, the collapse is observed above the equilibrium spreading pressure, which would suggest the formation of a heterogeneous metastable monolayer (Figures 2 and 8 in Gilby and Heymann^45^). This is unusual behavior and might be due to an experimental artifact. The overall trend of reduction of *S*_sp_ is possibly due to electrostatic screening of the surfactant head–head repulsion.

**Figure 10:**
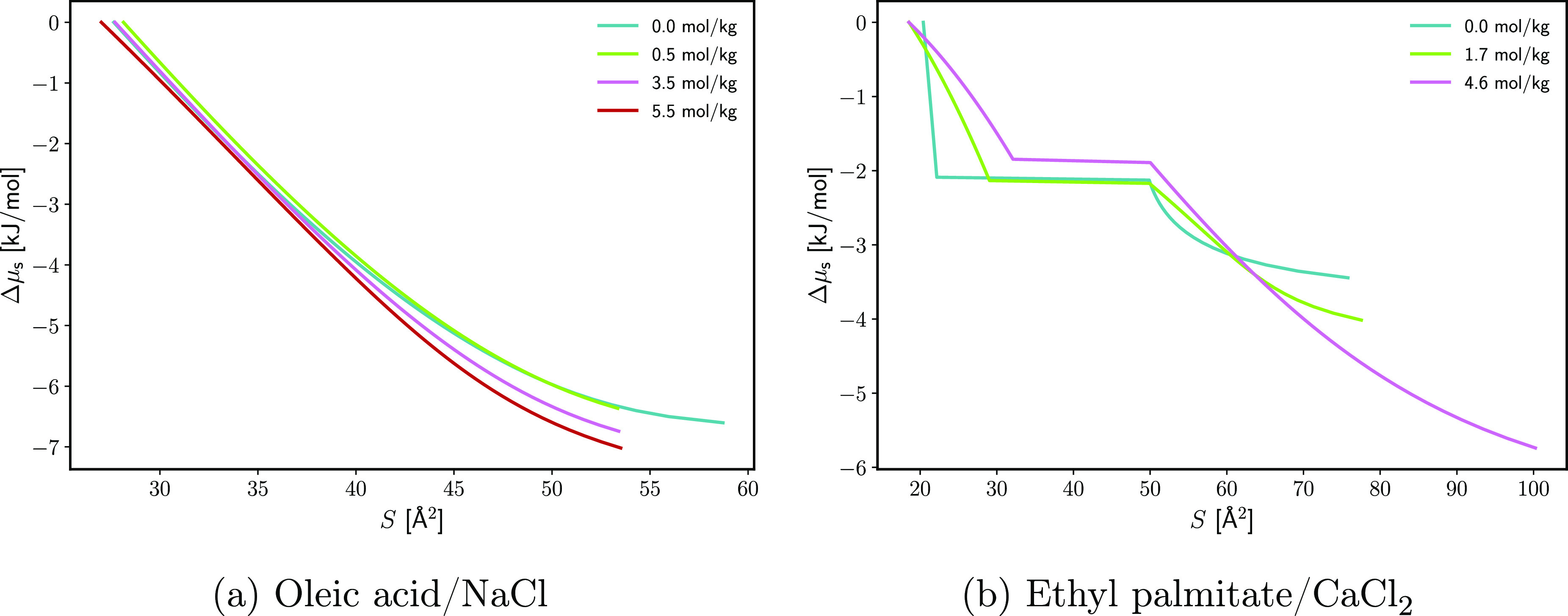
Change Δμ_s_ in the surface chemical potential relative to the reference state (equilibrium spread monolayer) as a function of the area per molecule *S* at different concentrations of electrolyte *C*_el,*m*_. Results were calculated through eq 23 using π(*S*) data from refs (42) and (45).

Once the reference points (π_sp_, *S*_sp_) are determined, the change in chemical potential of the surfactant Δμ_s_ can be calculated by integrating the π(*S*) isotherms (eq 23 and Figure 4). Details of the numerical procedure are given in section S2.5. Sample results are presented in Figure 10. The chemical potential difference Δμ_s_ can be expressed as
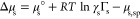
26where μ_s_°(*C*_el,*m*_) is the standard chemical potential of the surfactant on the surface and γ_s_(Γ_s_, *C*_el,*m*_) is the surface activity coefficient of the surfactant in the monolayer. Based on this formula, the electrolyte affects the chemical potential of the monolayer both through direct interaction between adsorbed surfactant molecules and the ions (via the μ_s_°(*C*_el,*m*_) term) and through an electrolyte-induced change in the intralayer lateral interaction and in the monolayer structure (via the γ_s_(Γ_s_, *C*_el,*m*_) term). In Figure 10, it can be seen that the electrolyte has a greater effect on the chemical potential in the dilute monolayer region. From the dilute region, we can judge for the salt effect on μ_s_°; the lowering of the chemical potential signifies stabilization of the system, i.e., attraction between the isolated surfactant molecules and the electrolyte solution. Similar conclusions have been drawn from MD simulations that show the electrolyte-induced stabilization of alcohol monolayers.^65^

For dense OA monolayers and Γ_s_ → Γ_s,sp_, the dependence of ln γ_s_ on *C*_el_ will be the main reason for the change in Δμ_s_ to *C*_el_. The observed effect of the electrolyte on slope ∂Δμ_s_/*∂S* in this region is marginal (Figure 10), which shows that the surfactant/surfactant interactions that control γ_s_ are barely altered by the electrolyte. The ions do not seem to penetrate between the surfactant molecules, as indicated by the small decrease in *S*_sp_, so they are unlikely to decrease the tail/tail van der Waals interactions. The OA tail is unsaturated, and the packed monolayer corresponds to a relatively large area per molecule, ∼28 Å^2^, controlled by the tail, compared to saturated carboxylic acids of area 18 Å^2^^66^ controlled by the −COOH headgroup. This corresponds to an ∼1 Å separation between the headgroups in a dense OA monolayer, which is (i) comparable with the bare ionic radii of the smaller cations and (ii) smaller than the Debye length of the solutions. In such a configuration, the electrolyte should be expected to have a screening effect on the head/head repulsion, i.e., stabilizing the monolayer and decreasing Δμ_s_. However, the effect appears to be small for OA. Unlike OA, the dense EP monolayer seems to be destabilized significantly by the electrolyte at high Γ_s_ (Figure 10). However, the slope ∂Δμ_s_/*∂S* after the phase transition could be influenced by the presence of a heterogeneous monolayer; i.e., this might be a nonequilibrium experimental artifact rather than an actual increase of Δμ_s_ with *C*_el_.

**Figure 11:**
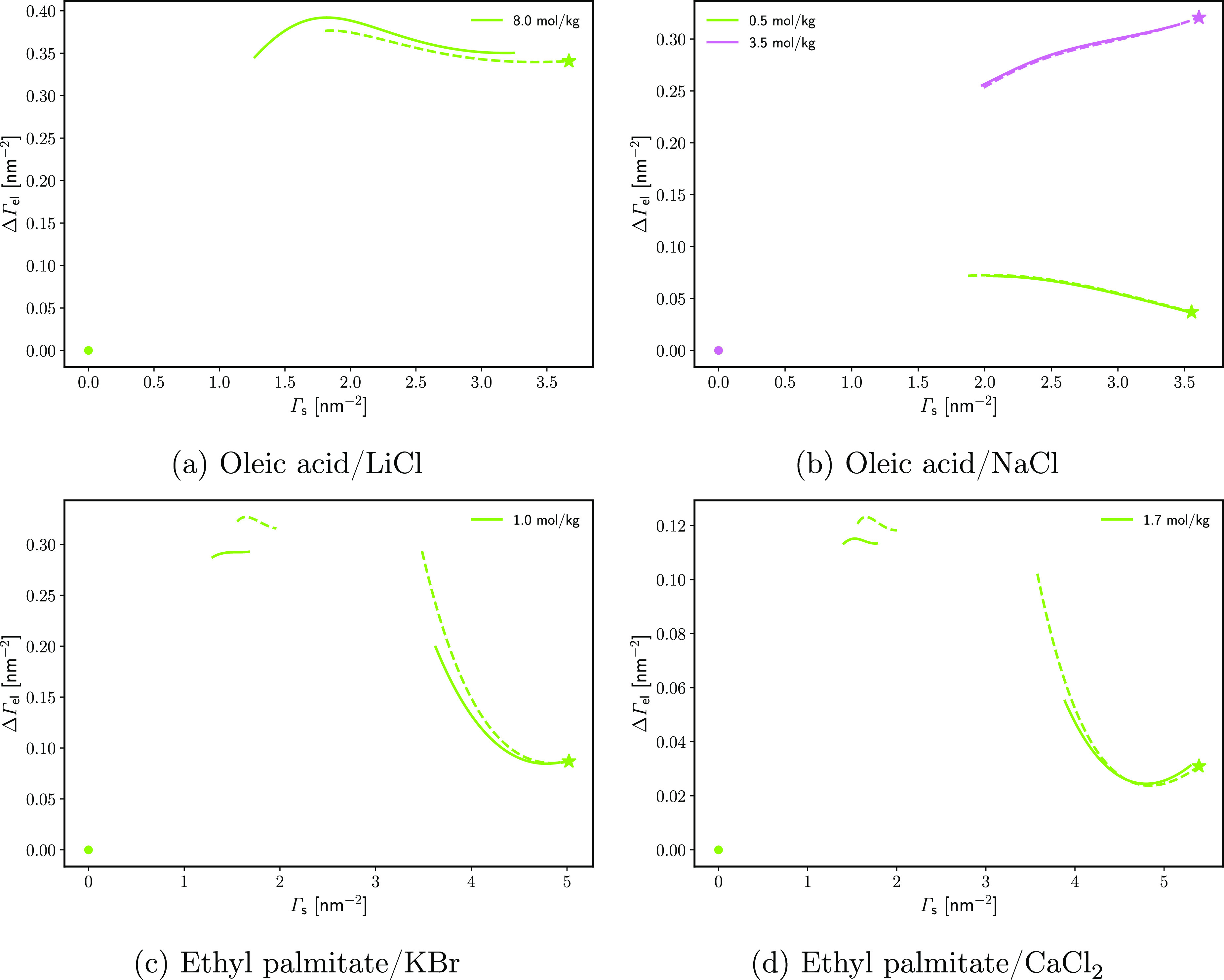
Increase in the surface excess of electrolyte with respect to water’s equimolecular plane on W|M compared to W|A ΔΓ_el_ as a function of the surfactant surface density, Γ_s_. The stars are calculated from the data for the equilibrium spreading pressure^42,44,45^ by using eq 19. The solid lines are calculated at constant π via eq 24; the dashed lines are calculated at constant Δμ_s_ via eq 19 using data from refs (42) and (45).

Finally, the monolayer-induced adsorptions ΔΓ_el_ were calculated as functions of the surfactant monolayer density Γ_s_ (Figure 11). By definition, at Γ_s_ = 0, ΔΓ_el_ is null. On the other end of the curves, the star symbols stand for the ΔΓ_el_ on a spread equilibrium monolayer, as calculated from the slope *∂π*_sp_/*∂p*_osm_ in the previous section. Between these limits, the two ΔΓ_el_(Γ_s_) curves are calculated according to eq 19 (the approach of Pankratov-Frumkin) and 24 (the isobaric route); the results agree very well. The most important feature of these curves is that the monolayer-induced adsorption of electrolyte changes nonmonotonously with the increase in the monolayer density. This confirms our previous findings for dodecanol monolayers.^27^ At intermediate surface coverage, there is a maximum of ΔΓ_el_ for all studied systems, with the exception of 3.5 mol/kg NaCl on OA. A similar “squeezing out” effect has been observed previously on lipid monolayers;^46,48^ however, it has not been quantified or explained. Note that the shift of the water equimolecular plane due to the presence of headgroups in the surface layer cannot explain this trend; the “osmotic” effect (i.e., the second term in eq 25) is nearly linear with respect to Γ_s_ and hence the shape of ΔΓ_el_ is characteristic of  as well. Hence, direct ion–surface interactions must be present.

### Analysis of Data for Δ*V* Potential

**Figure 12:**
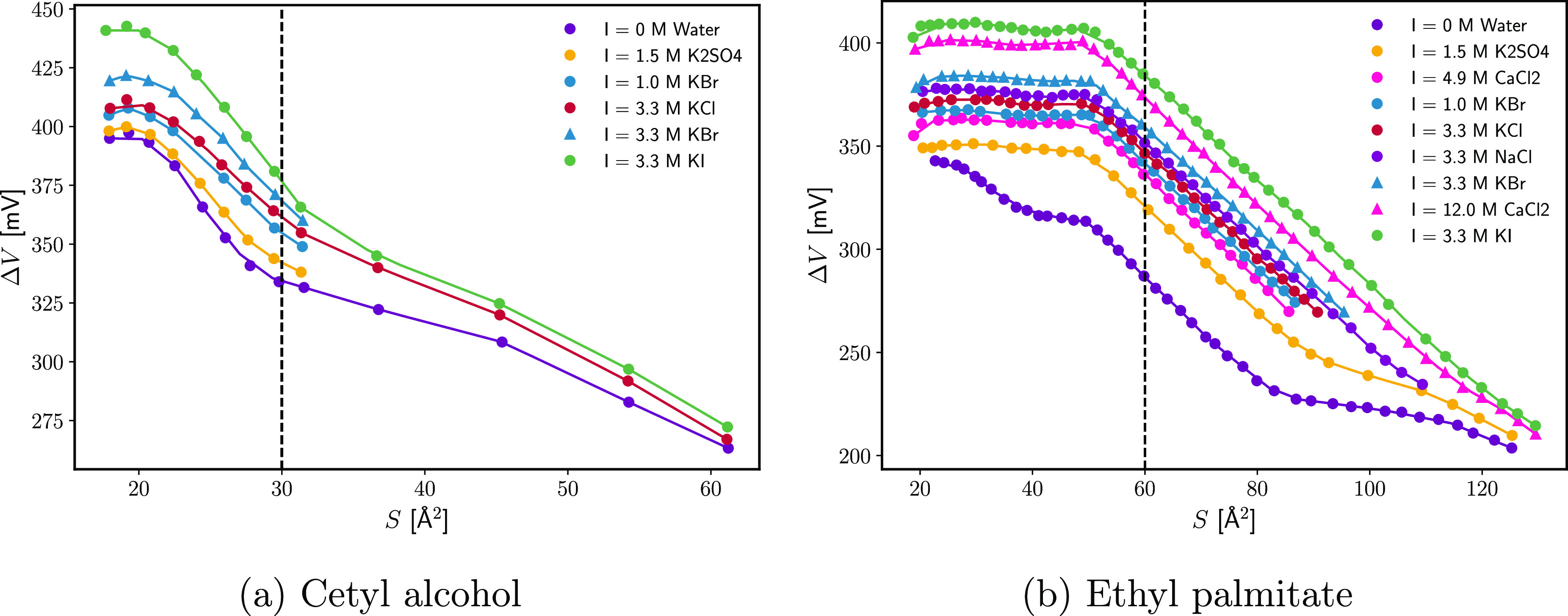
Change of the Volta potential Δ*V* as a function of the area per surfactant molecule *S* at different types and concentrations of electrolyte. Data were digitized from ref (42).

In this section, the change in Volta potential Δ*V* upon spreading ethyl palmitate and cetyl alcohol (CA) over aqueous electrolytes is analyzed (Figure 12). The data are from Pankratov^42^ at 20 ± 2 °C. By definition, Δ*V* is the change in the surface potential upon spreading of a monolayer
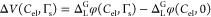
27where  is the potential difference between the liquid and gas phase. The potential  is simply the χ(*C*_el_) potential of the electrolyte solution. The difference Δχ ≡ χ(*C*_el_) – χ_0_ is a measurable quantity known for many electrolytes.^67−69^ However, no direct method can measure the surface potential of pure water, , and its estimates vary greatly; we will use the value −90 mV for it.^6^ The potential  in the presence of a monolayer could be divided into two contributions: the electric double layer (EDL) potential ϕ_DL_ from the distribution of free charge near the surface and the dipolar potential Γ_p_/ε_0_ from the total normal dipole moment of the surface Γ_p_.

The specifically adsorbed surface dipole moment *P*_s_ (due to the orientation of the headgroups and, to a lesser degree, the surface water) produces an electric field that extends to a few angstroms around the surface.^70^ This field polarizes the solvent in the direction opposite to that of the headgroups. Thus, a dipole double layer (DDL) is formed (Figure 11 in ref (40)), consisting of an adsorbed part from the normal surface dipole moment *P*_s_ and a diffuse part *P*_diff_ made of oppositely polarized molecules in the adjacent media. According to the quadrupolar electrostatics, the total surface dipole moment Γ_p_ = *P*_s_ + *P*_diff_ can be related to the specifically adsorbed dipole as
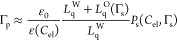
28where  and  are the quadrupolar lengths in the water and the “oil” (the layer of surfactant tails) mediums, respectively.^70^ We can assume that the quadrupolar lengths *L*_q_ do not change much with the electrolyte concentration (eq in ref (71)). However,  of the layer of hydrocarbon tails varies with the density of the monolayer. Using eq 28, we can express the Volta potential of the monolayer through *P*_s_, ϕ_DL_, and Δχ:
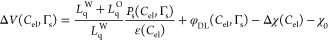
29This formula highlights that the measured Δ*V* potential reflects the change in both ion adsorption (through the EDL potential ϕ_DL_) and the orientation of the surfactant headgroups and water in the surface layer (through the specifically adsorbed normal surface dipole *P*_s_). Moreover, in concentrated electrolytes, Δ*V* is significantly affected by the bulk-ion-specific value of ε(*C*_el_). As the concentration of the electrolyte is increased, the ability of the solvent to counteract the monolayer dipole moment is reduced, leading to Δ*V* potentials higher in absolute value.

**Figure 13:**
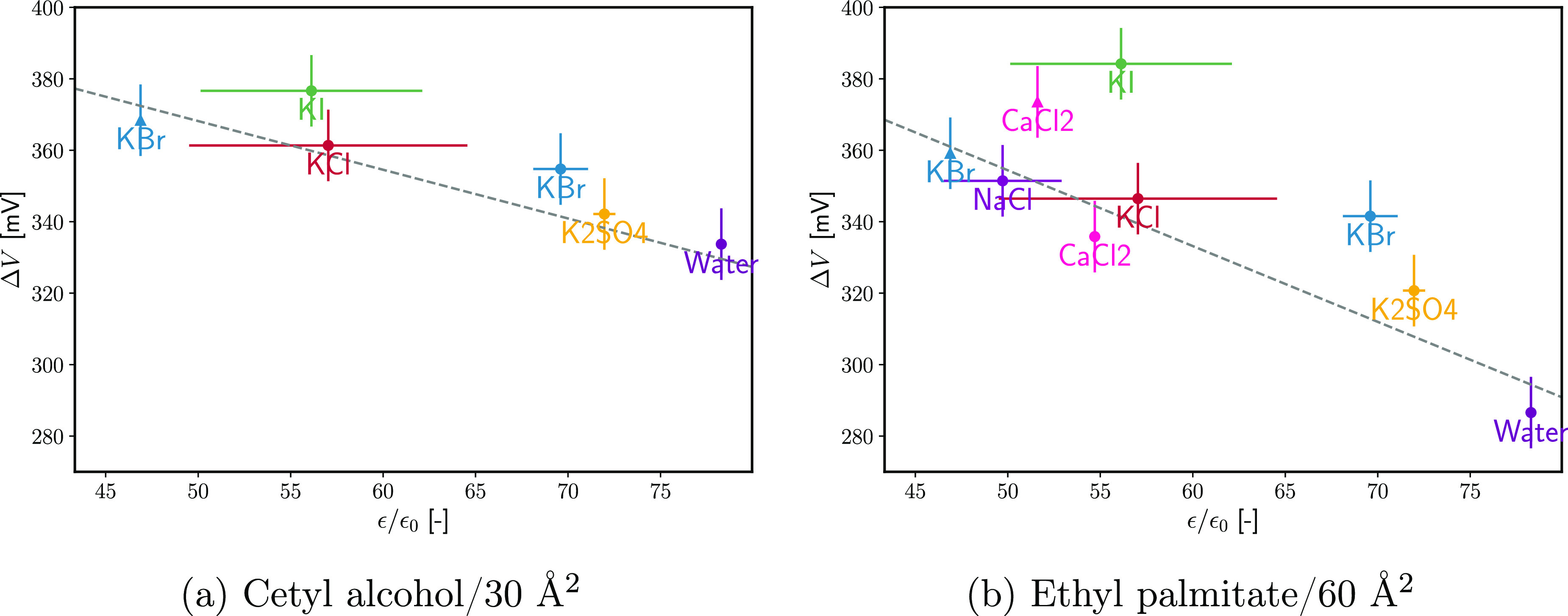
Change of the Volta potential Δ*V* at a fixed monolayer density as a function of the relative dielectric permittivity of the electrolyte solution ε. The dashed gray lines shown are to guide the eye only. The Δ*V* potentials are from ref (42). The ε data are from refs (72−75). The ε error bars are determined by the uncertainty in the data for ε from the different sources where available. The Δ*V* is assumed to be reproducible within ±10 mV.^77^

On the W|A surface without surfactant, the largest contribution to the variation of the Δχ potential with *C*_el_ is, in fact, from the variation of the bulk dielectric permittivity ε.^6^ The addition of electrolyte decreases the collective dielectric response of the subphase to the field of the adsorbed dipoles and, as such, reduces the dipole/dipole correlation, resulting in a more polarized interface (i.e., |*P*_diff_(*C*_el_, 0)| < |*P*_diff_(0, 0)|). To test if Δ*V* behaves similarly, Figure 13 shows the Δ*V* values at a constant monolayer density as a function of the dielectric permittivity of the solution (taken from refs (72−75).). It can be seen that the two correlate quite well. This suggests that bulk ε plays a large role in the change of the Volta potential with *C*_el_. The most prominent outlier from the correlation is the I^–^ salt, which produces very positive Δ*V* potentials. I^–^ is a sticky ion and is expected to adsorb on the interface. However, the resulting negative ϕ_DL_ appears to be largely canceled by the respective negative Δχ (eq 29), and the Δ*V* is instead more positive than expected from the correlation. Frumkin and Pankratov explain this with the increase in the intrinsic surface dipole moment *P*_s_, as a result of surfactant–ion interactions. One might expect a similar effect from Br^–^, as it is on the border between surface-inactive and sticky ions. More recent studies on phospholipid monolayers have also ascribed large potential differences to ion-induced rearrangement rather than an EDL with unreasonably high ion adsorptions.^50,76^

For CA, the maximum increment ΔΔ*V* of the volta potential upon addition of electrolyte is about 40 mV, while for EP it is around 80 mV. In light of eq 29, this difference could be the result of two effects: (i) the surfactant dipole moment of the ester headgroup changes with the addition of electrolyte due to ion-induced tilting, more than that of the alcohol headgroup; (ii) the electric double layer potential changes more in the presence of one of the surfactants than the other. Both effects point to a specific interaction between the headgroups and the ions.

Unfortunately, even if ε(*C*_el_), Δχ(*C*_el_), and  are all known for a given system, eq 29 still relates two unknowns, *P*_s_ and ϕ_DL_, to a single measured characteristic, Δ*V*. In the literature, it is common to assume that the total adsorbed dipole Γ_p_ is independent of *C*_el_ and then to use a variant of eq 29 to determine ϕ_DL_. However, eq 28 clearly shows that Γ_p_ is a strong function of *C*_el_, through concentration-dependent permittivity ε. In our previous work,^27^ we assumed instead that the specifically adsorbed normal dipole *P*_s_ does not depend on *C*_el_, at least not for the supposedly surface-inactive NaCl on alkanol monolayers. This assumption allows the double-layer potential to be extracted. If it is true that the specifically adsorbed dipole does not change with the addition of electrolyte, then one can solve eq 29 for ϕ_DL_:
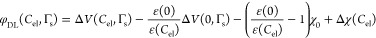
30However, the assumption *P*_s_(*C*_el_, Γ_s_) = *P*_s_(0, Γ_s_) cannot hold true if there is a significant interaction between the ion and the monolayer. Another complication is that, for water, the diffuse dipole and the adsorbed dipole layers overlap (as their thicknesses are similar). This overlap makes *P*_s_ an effective quantity, with an absolute value greater than the actual density of adsorbed dipoles.^70^

**Figure 14:**
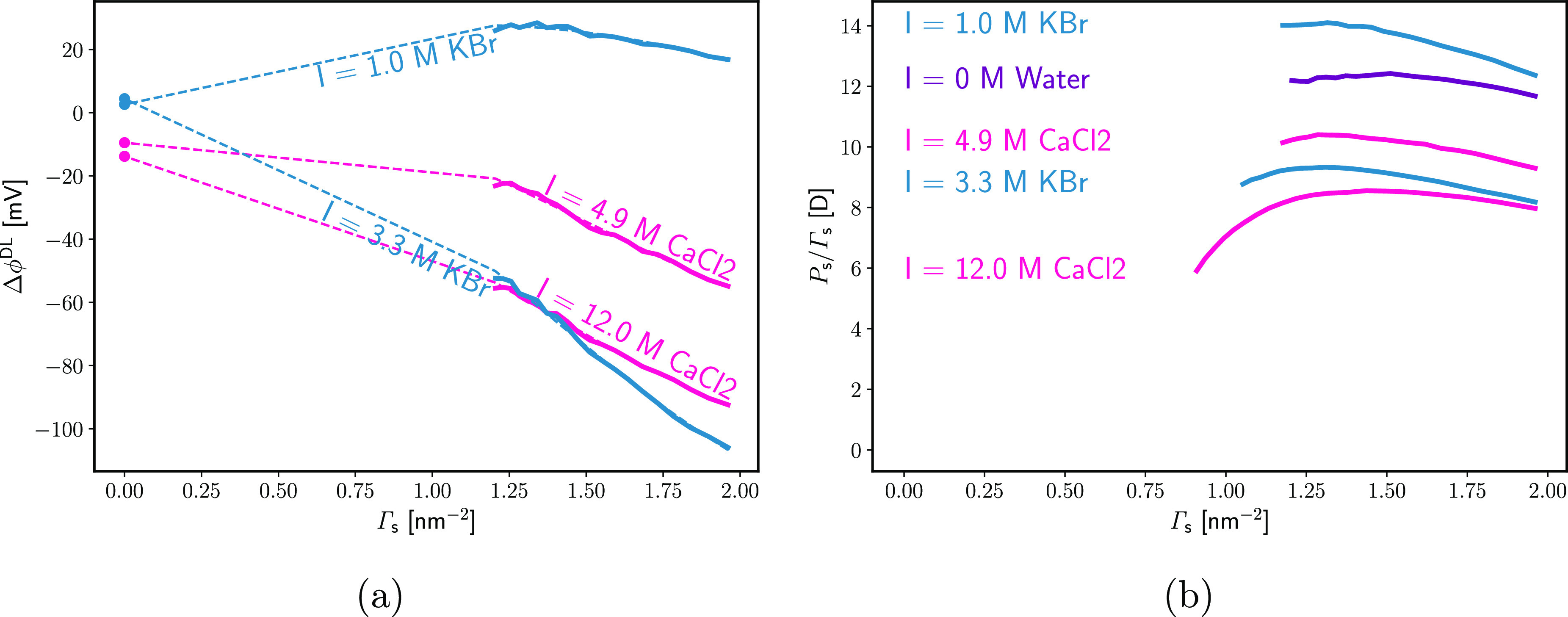
(a) Electric double layer potential Δϕ_DL_ as a function of the EP monolayer density Γ_s_ under the assumption for the electrolyte-independent specifically adsorbed surface dipole moment (eq 30). (b) Effective specifically adsorbed normal dipole per adsorbed surfactant molecule *P*_s_/Γ_s_ as a function of the EP monolayer density Γ_s_ under the assumption of a monolayer-independent electric double layer potential (eq 31). The Δχ(*C*_el_) potentials were taken from refs (67) and (69). χ_0_ is assumed to be equal to −90 mV.

To test the assumption, the electric double layer potentials are computed from eq 30 for KBr and CaCl_2_ on EP monolayers, as shown in Figure 14. To avoid complications from the heterogeneity of the monolayer, only the liquid expanded state is considered. For 1 M KBr, the computed ϕ_DL_ is positive and 10–20 mV higher than the one following from eq 8. This potential agrees roughly with the value of ΔΓ_el_ calculated in Figure 11: the monolayer-induced adsorption of KBr is about 0.3 nm^–2^ in the liquid expanded phase, which corresponds to an additional surface charge density *e*ΔΓ_el_ = 0.05 C/m^2^. The linear Gouy equation then predicts a potential increase of Δϕ_DL_ = *e*ΔΓ_el_*L*_D_/ε ≈ 20 mV, under the assumption that K^+^ is the potential-determining (closer to the interface) ion. However, at 3.3 M KBr, the computed ϕ_DL_ changes sign, suggesting that either Br^–^ adsorbs strongly or the assumption of constant *P*_s_ fails. While a surge of the adsorption of Br^–^ above 2 M is not at all impossible and is evident also from tensiometric data at neat W|A and W|O, an absolute value of ϕ_DL_ of [[[*x*02011]]]100 mV is highly unlikely for potential screened by the 3.3 M electrolyte. Therefore, a significant change in *P*_s_ seems to be taking place. Similarly, the calculated ϕ_DL_ for CaCl_2_ are too negative. If we assume that the specific adsorption of CaCl_2_ at 1.65 M is 0.10 nm^–2^ (Figure 11) and is entirely due to the Cl^–^ ions, then the specific adsorption of potential-determining ions is 0.2 nm^–2^, corresponding to surface charge of −0.03 C/m^2^ and ϕ_DL_ dropping by 5–10 mV, as compared to −30 mV from eq 30. Thus, the assumption of constant *P*_s_ might hold for 1 M KBr only but not for the other three systems in Figure 14.

We therefore investigate another limit, where we assume that ϕ_DL_ in eq 29 is not significantly affected by the monolayer and follows the predictions of the MS model, eq 8. This allows eq 29 to be solved for *P*_s_:
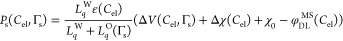
31The utilization of this formula requires the value of the ratio . Based on the quadrupolar cavity model of *L*_q_(78) and assuming that the quadrupolarizability of the EP liquid expanded layer is similar to that of dense CH_4_ under high pressure, this ratio is roughly on the order of 1/2 (and increases as the density of the liquid expanded phase decreases).

The surface polarization calculated from eq 31, with , is presented in Figure 14 as the dipole moment per adsorbed EP molecule, *P*_s_/Γ_s_. If the assumption for ϕ_DL_ being independent of Γ_s_ holds true, then the dipole moment calculated from eq 31 suggests that the addition of 1 M KBr to water increases *P*_s_/Γ_s_ by about 1 to 2 D. We rather expect that the addition of electrolyte depolarizes (disorganizes) the specifically adsorbed dipole layer (mostly through the contribution of surface water). Therefore, for 1 M KBr, the first investigated limit (constant *P*_s_ and increasing ϕ_DL_ due to the specific adsorption of K^+^, eq 30) seems closer to the truth. In contrast, for 3.3 M KBr, *P*_s_/Γ_s_ drops significantly compared to that for water. For CaCl_2_, there is a steady decrease in *P*_s_/Γ_s_ as the electrolyte concentration increases. Thus, the main reason for the change in Δ*V* seems to be the change in the orientation of specifically adsorbed dipoles. At 4 M CaCl_2_, a significant drop in the computed *P*_s_/Γ_s_ is observed at Γ_s_ < 1 nm^–2^. This is probably the result of the decreased density of the hydrocarbon layer in this range, leading to a drop in  and  approaching 1. Of course, the observed Δ*V* is most likely the result of both a drop in *P*_s_/Γ_s_ and a specific adsorption of ions affecting the ϕ_DL_. However, our analysis suggests that in many cases assuming a constant ϕ_DL_ could be a better approximation than assuming a constant *P*_s_.

## Conclusions

The behavior of surface-inactive electrolytes on W|A and W|O interfaces is controlled by hydration and image forces, with little direct ion specificity, even at very high concentration.^6,8,59^ The same electrolytes show far more complex and clearly directly ion-specific interactions with proteins and other colloid systems.^4,7^ In this work, we set out to analyze the ion-specific effect in a system of intermediate complexity: uncharged spread monolayers. For this analysis, we use a new methodology based on the old idea of Pankratov and Frumkin to use the equilibrium spread monolayer as a reference state to determine the electrolyte adsorption and the recent quadrupolar theory of the dipole double layer^70^ to investigate the surface potential of the monolayer in the presence of electrolyte. From this analysis, a fascinatingly complex picture emerges.

To characterize quantitatively the ion–monolayer interaction, we introduce and calculate a new variable—the monolayer-induced electrolyte adsorption ΔΓ_el_—that compares the surface concentration of electrolyte on a surface with and without a monolayer. Upon addition of a surface-active substance to the electrolyte|air interface, the uncharged monolayer tends to increase the adsorption of the electrolyte (Figures 6 and 11). A major reason for this extra adsorption is purely osmotic: the surfactant headgroups expel water from the interface, resulting in a shift of the water equimolecular interface toward the bulk solution (corresponding to an effective ion adsorption, eq 16). This contribution is surfactant-specific (through the polar group volume, *V*_s_). A second significant contribution seems to be in play which is due to specific ion–monolayer interactions. These interactions can be attractive or repulsive (Figure 7), depending on the type of constituent ions and the density and nature of the monolayer. Thus, for the monolayer-covered water surface, a direct surface ion specificity is exhibited even by surface-inactive ions.

We developed the methodology to calculate the monolayer-induced adsorption building upon the work of Frumkin and Pankratov. The method combines spreading pressure and surface pressure to obtain area data. The route to calculating the excess electrolyte proposed by Frumkin and Pankratov has been realized only recently^27^ for the example of alcohol monolayers. Here, we developed a new route based on numerical differentiation under a constant surface pressure (Figure 4). Both methods require the intermediary calculation of the change in the surfactant chemical potential compared to the reference upon expansion, which on its own is a powerful tool for studying ion/surfactant interactions (Figure 10). For instance, using this potential, we have shown that dilute uncharged monolayers are stabilized by salts (lower chemical potential), suggesting an attractive interaction between the isolated surfactant molecule and the ions from the solution.

Our analysis of Δ*V* potential data for the monolayers shows that the increase in Δ*V* at a constant monolayer density with the addition of electrolyte is well correlated with the variation of bulk dielectric permittivity ε (Figure 13) and therefore might not be very informative with respect to the state of the surface (i.e., an indirect surface ion-specific effect, similar to Δχ at W|A^6^). However, the Δ*V* data for some electrolytes, such as KBr and CaCl_2_, also show direct interaction. The interaction results in a simultaneous change in the orientation of the adsorbed dipoles and the EDL potential. For most studied systems, the common assumption for electrolyte-independent surface dipole moment is not correct. It actually appears that it is more accurate to assume a monolayer-independent EDL potential or accept that both quantities change.

While we abstain from discussing the microscale nature of the ion-specific interactions behind the complex behavior of ΔΓ_el_ and Δ*V*, some important conclusions emerge. One is that the solvation potential appears to be an important factor. For example, the tendency for the desorption of Li^+^ and Mg^2+^ and for little overall interaction of Rb^+^ and Ba^2+^ with a dense OA monolayer (Figure 7) can be explained with the smaller ions being better solvated by water and the larger ones not distinguishing water and −COOH. This may be the reason that the adsorption of Li^+^ (unsolvated by the surfactant) does not change much for oleic acid and diethyl sebacate, while Rb^+^ that is attracted by the acid appears to be expelled from the ester monolayer (Figure 6). This solvation effect may also be the reason for the maximum in ΔΓ_el_ at intermediate monolayer densities: it is reasonable to assume that ions are best solvated by the monolayer at a specific mean distance between the polar headgroups, allowing geometrically for the best coordination with the cations or optimal hydrogen bonding with the anions. These hypotheses, however, have to withstand additional scrutiny.

## Supporting Information Available

Table listing the source of all used data; description of numerical manipulations, including general formulas, fitting parameters, specific algorithms, and error estimation; estimation of the H^+^ adsorption on acid monolayers; a limiting nonspecific model for the excess depletion layer thickness; and a dielectric multilayer model for the dielectric permittivity and its application to calculating partial molar volumes of surfactants (PDF)
