# Supplementary material for: Interactions between Small Inorganic Ions and Uncharged Monolayers on the Water/Air Interface
Source: J Phys Chem B. 2023 Mar 17;127(12):2801–17. doi: 10.1021/acs.jpcb.2c08019 (PMC10068745; doi:10.1021/acs.jpcb.2c08019)
Supplement: Supplementary file 1 — jp2c08019_si_001.pdf [file jp2c08019_si_001.pdf]

Supporting Information: Interactions between Small Inorganic Ions and Uncharged Monolayers on the Water/Air Interface

Boyan Peychev and Radomir I. Slavchov*

Queen Mary University of London, School of Engineering and Materials Science, Mile End Road, London E1 4NS, United Kingdom

E-mail: r.slavchov@qmul.ac.uk

S1 Experimental data

Table S1: References to the original sources of the experimental data used in the paper.

Salt	V_{el}	S_ρ	$y_{\text{el,m}}(C_{\text{el,m}})$	$\varepsilon(C_{\text{el,M}})$		
KF	S1	S1	S2	-		
LiCl	S1	S1	S2	-		
NaCl	S1	S1	S2	S3–S5		
KCl	S1	S1	S2	S3,S5–S7		
RbCl	S1	S1	S2	-		
KBr	S1	S1	S2	S3,S5		
KSCN	S8	Detherm	Aspen	-		
KI	S1	S1	S2	S3,S5,S7		
$MgCl_2$	S8	S1	S9	-		
$CaCl_2$	S1	S1	S10	S5		
$BaCl_2$	S1	S1	S9	-		
	Oleic acid			Ethyl palmitate		
	$\pi_{\text{sp}}(C_{\text{el,m}})$	$\pi(C_{\text{el,m}}, S)$	$\Delta V(C_{\text{el,m}}, S)$	$\pi_{\text{sp}}(C_{\text{el,m}})$	$\pi(C_{\text{el,m}}, S)$	$\Delta V(C_{\text{el,m}}, S)$
LiCl	S11	S11	-	-	-	-
NaCl	S11	S11	-	-	S12	S12
KCl	S11	S11	-	-	S12	S12
NH_4Cl	S11	-	-	-	-	-
KBr	S11	S11	-	S13	S12	S12
KSCN	S11	-	-	-	-	-
KI	-	-	-	S13	S12	S12
$MgCl_2$	S11	S11	-	-	-	-
$CaCl_2$	S11	S11	-	S13	S12	S12
$BaCl_2$	S11	S11	-	-	-	-
	Diethyl sebacate			Cetyl alcohol		
	$\pi_{\text{sp}}(C_{\text{el,m}})$	$\pi(C_{\text{el,m}}, S)$	$\Delta V(C_{\text{el,m}}, S)$	$\pi_{\text{sp}}(C_{\text{el,m}})$	$\pi(C_{\text{el,m}}, S)$	$\Delta V(C_{\text{el,m}}, S)$
KF	S14	-	-	-	-	-
LiCl	S14	-	-	-	-	-
NaCl	S14	-	-	-	-	-
KCl	S14	-	-	-	-	S12
RbCl	S14	-	-	-	-	-
KBr	S14	-	-	-	-	S12
KI	S14	-	-	-	-	S12
KSCN	S14	-	-	-	-	-

S2 Numerical calculations

S2.1 Activity coefficients

The conversion between molal activity $a_{\mathrm{el,m}}$ and molality $C_{\mathrm{el,m}}$ is done according to the relation

$$a_{\mathrm{el,m}} = \gamma_{\mathrm{el,m}} C_{\mathrm{el,m}} \tag{S1}$$

The molal based activity coefficients of the electrolytes were calculated with an empirically corrected Debye–Hückel law

$$\lg \gamma_{\mathrm{el,m}} = -\frac{|z_+ z_-| A \sqrt{I_{\mathrm{m}}}}{1 + B\sqrt{I_{\mathrm{m}}}} + \sum_{i=1}^{6} \beta_i I_{\mathrm{m}}^i, \tag{S2}$$

where z_i are the ion's charges and I_{m} is the molal ionic strength of the solution

$$I_{\mathrm{m}} = \frac{1}{2} \sum z_i^2 C_{\mathrm{m},i}. \tag{S3}$$

The coefficient A is calculated according to[S15]

$$A = \frac{e^3 \lg \mathrm{e}}{4\pi} \sqrt{\frac{N_{\mathrm{a}} \rho_{\mathrm{w}}}{2k^3 T^3 \epsilon^3 \epsilon_0^3}}, \tag{S4}$$

where e is the elementary charge, N_{a} is Avogadro's constant, ϵ_0 is the dielectric permittivity of vacuum and ρ_w is the density of water. The electrolyte specific coefficients B and β_i were collected from the literature (refer to table S1). The values of the parameters are presented on table S2.

Table S2: Bulk properties of electrolyte solutions

Electrolyte	V_{el} [m^3/mol]	S_ρ [M$^{-3/2}$]	B [mol/kg$^{-1/2}$]	β_1 [mol/kg^{-1}]	β_2 [mol/kg^{-2}]	β_3 [mol/kg^{-3}]	β_4 [mol/kg^{-4}]	β_5 [mol/kg^{-5}]	β_6 [mol/kg^{-6}]	$C_{el,m,max}$ [mol/kg][a]
KF	6.6e-06	0.00335	1.29	0.027845	0.005	-0.0002531	2.679e-06	0	0	17.5
LiCl	1.7e-05	0.001488	1.305	0.11603	-0.007773	0.002928	-0.00032	1.4068e-05	-2.2498e-07	19.2
NaCl	1.64e-05	0.002153	1.4495	0.020442	0.005793	-0.000289	0	0	0	6.1
KCl	2.652e-05	0.002327	1.295	7e-05	0.003599	-0.000195	0	0	0	5
RbCl	3.187e-05	0.002219	1.1439	0.00081	0.003246	-0.00022672	0	0	0	7.8
NH_4Cl	3.598e-05	0.00145	1.325	-0.004579	0.005271	-0.000706	2.8e-05	0	0	7.4
$MgCl_2$	1.44e-05	0.00968	1.63348	0.056385	0.005995	-0.000152	0	0	0	5
$CaCl_2$	1.825e-05	0.00599	1.61049	-0.003593	0.0142574	-0.000729	1.407e-05	-9.081e-08	1.809e-24	10.5
$BaCl_2$	2.36e-05	0.00483	1.57654	0.015872	0.003311	0.000151	-4.6e-05	0	0	1.8
KBr	3.373e-05	0.001939	1.35	0.003975	0.003945	-0.0003	0	0	0	5.5
KI	4.536e-05	0.001556	1.381	0.025012	-0.000117	0	0	0	0	4.5
KSCN	5.66e-05	0.001892	1.3	-0.003981	0.002753	-0.000446	3.122e-05	-1.045e-06	1.358e-08	22.9826
K_2SO_4	3.228e-05	0.01811	1.08046	-0.0288895	-0.0006598	0	0	0	0	0.7

[a] The parameters B and β_i are determined by interpolating experimental data up to concentrations $C_{el,m,max}$.

S2.2 Molarities

The conversion between molarities $C_{\mathrm{el,M}}$ and molalities of the solutions was done using

$$C_{\mathrm{el,m}} = \frac{C_{\mathrm{el,M}}}{\rho_{\mathrm{w}}(1 - V_{\mathrm{el}}C_{\mathrm{el,M}} - S_\rho I_{\mathrm{M}}^{3/2})}, \tag{S5}$$

where V_{el} is the partial molar volume of the electrolyte and S_ρ is an empirical parameter compensating for solute-solvent interactions. Both can be found on table S2.

S2.3 Osmotic pressure

The osmotic pressure is by definition

$$p_{\mathrm{osm}} = kT\rho_{\mathrm{w}}\gamma_{\mathrm{w,x}}C_{\mathrm{el,m}}, \tag{S6}$$

where

$$\gamma_{\mathrm{w,x}} = \frac{1}{C_{\mathrm{el,m}}}\int_0^{C_{\mathrm{el,m}}} C_{\mathrm{el,m}}\frac{\mathrm{d}\ln a_{\mathrm{el,m}}}{\mathrm{d}C_{\mathrm{el,m}}}\mathrm{d}C_{\mathrm{el,m}} \tag{S7}$$

Using equation S2, $\gamma_{\mathrm{w,x}}$ can be found analytically

$$\begin{aligned}\gamma_{\mathrm{w,x}} = \ln 10\frac{|z_+z_-|A\sqrt{k_{\mathrm{is}}}}{B^2k_{\mathrm{is}}C_{\mathrm{el,m}}}\left(\sqrt{C_{\mathrm{el,m}}}\left(B\sqrt{k_{\mathrm{is}}}\sqrt{C_{\mathrm{el,m}}} - 2\right) + \frac{2\ln\left(1 + B\sqrt{k_{\mathrm{is}}}\sqrt{C_{\mathrm{el,m}}}\right)}{B\sqrt{k_{\mathrm{is}}}}\right) \\ - \ln 10\frac{|Z_+Z_-|A\sqrt{k_{\mathrm{is}}}\sqrt{C_{\mathrm{el,m}}}}{1 + B\sqrt{k_{\mathrm{is}}}\sqrt{C_{\mathrm{el,m}}}} + \ln 10\sum_{i=1}^{6}\frac{i}{i+1}\beta_j k_{\mathrm{is}}^i C_{\mathrm{el,m}}^i + 1,\end{aligned} \tag{S8}$$

where $k_{\mathrm{is}} = I_{\mathrm{m}}/C_{\mathrm{el,m}}$.

S2.4 Differentiation of $\Delta\pi_{\mathrm{sp}}$

Figure S1a compares the results for the monolayer-induced adsorption of electrolyte $\Delta\mathit{\Gamma}_{\mathrm{el}}$ calculated by explicit interpolation (solid line) and three point finite difference (points). As it can be seen the two methods are in agreement. Within the collected data, most systems

have a reasonable number of experimental points. There are a number of system where only one point is present. For those systems, the only possible interpolation is with a linear dependence. Similarly, only two point finite difference can be used, with the origin. In that case, the two methods are identical. In between, there are a few systems, like LiCl on ES, where the number of points is low and the results can vary depending on the chosen method of differentiation. This is illustrated on figure S1b. For RbCl with only one experimental point, the two methods give identical results. For KCl, with two experimental points, the two methods happen to deviate from each other significantly. For LiCl and NaCl, with three and four experimental points, the interpolating method gives an averaged line between the finite difference points.

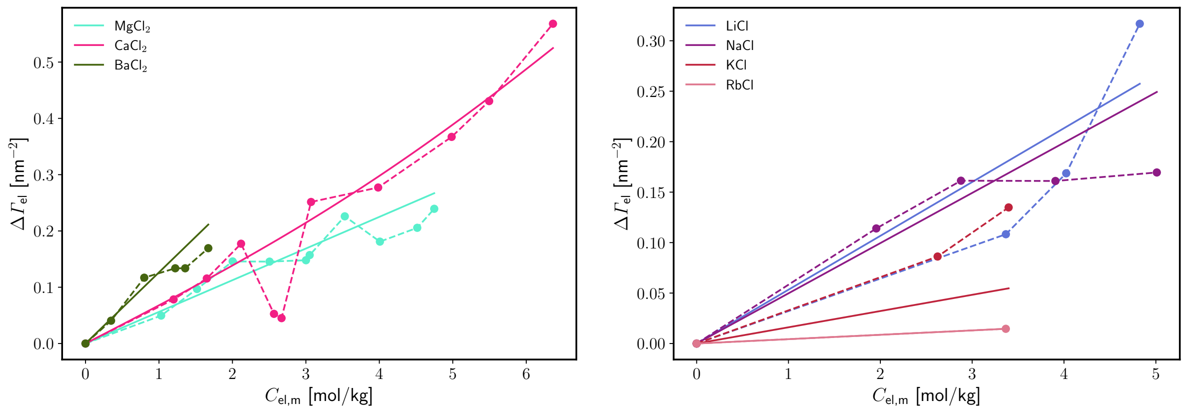

(a) Oleic acid

(b) Diethyl sebacate

Figure S1: Comparison of monolayer-induced adsorption of electrolyte $\Delta\Gamma_{el}$ calculated by differentiating an explicitly interpolated function (solid lines) and differentiating numerically with two/three point finite difference (points).

To make the results easier for comparison, the approach, opted for here, was to interpolate the $\Delta\pi_{sp}(p_{osm})$ data with a polynomial before differentiating analytically. The choice of explicit interpolation over finite point difference is motivated by the smoothing of the experimental noise, characteristic for the method. The interpolation was done with a $1^{\text{-st}}$ or $2^{\text{-nd}}$ degree polynomial, with a fixed intercept $\Delta\pi_{sp}(0) = 0$. A higher than $2^{\text{-nd}}$ degree polynomial was excluded from the consideration. A binome was chosen only if:

1. There are more than three experimental points. That is to impose at least one degree of freedom for the fit.

2. The standard deviation s of the linear fit is larger than 0.1 mN/m. Deviations of 0.1 mN/m or less are comparable to the experimental uncertainty. In that case raising the degree of the polynomial risks overfitting the experimental noise.

3. The standard deviation of the quadratic fit is 20% or more lower than the standard deviation of the linear fit. This is to ensure that there is a reasonable improvement to be gained at the cost of more free parameters.

The fits are compared to the experimental data graphically on figure S2. The fitting parameters and standard deviations are presented in table S3. The relevant fitting function is:

$$\Delta\pi_{\mathrm{sp}} = a_2 p_{\mathrm{osm}}^2 + a_1 p_{\mathrm{osm}} \tag{S9}$$

From the comparison of the calculated $\Delta\mathit{\Gamma}_{\mathrm{el}}$ using the interpolated lines and with finite point difference, the error of the method is evaluated (see S1a). The vertical distance between the points and lines was averaged over all electrolytes and points to give an error of 0.026 nm^{-2}. That is quite small compared to the characteristic monolayer-induced adsorptions $\Delta\mathit{\Gamma}_{\mathrm{el}}$ calculated. However, it should be kept in mind that the average error is weighted heavily by well resolved data, e.g. $CaCl_2$ on OA. The actual error is much larger for systems with only a few data points, e.g. KCl on ES.

S2.5 Calculating $\Delta\mu_{\mathrm{s}}$

To integrate the isotherms, they were first interpolated explicitly. The OA isotherms were described as a polylog function

$$\mathrm{S}_{\mathrm{LE}}(\pi) = S_{\mathrm{ref}} - c_1 \ln\left(\frac{\pi}{\pi_{\mathrm{ref}}}\right) - c_2 \ln^2\left(\frac{\pi}{\pi_{\mathrm{ref}}}\right) - c_3 \ln^3\left(\frac{\pi}{\pi_{\mathrm{ref}}}\right) \tag{S10}$$

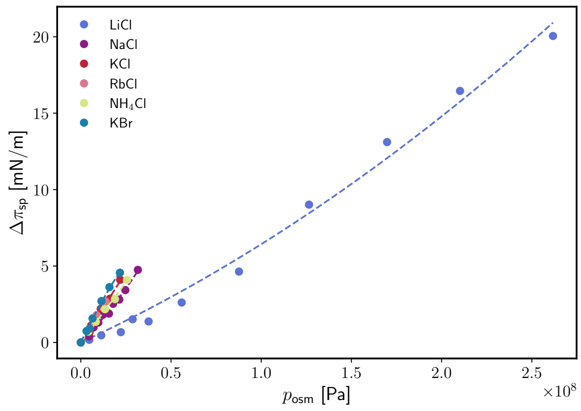

(a) Oleic acid/1:1 electrolytes

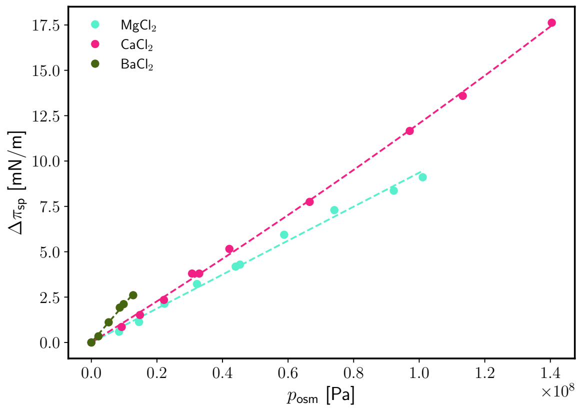

(b) Oleic acid/1:2 electrolytes

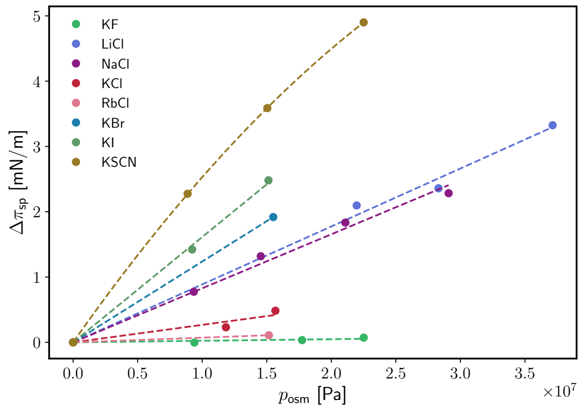

(c) Diethyl sebacate

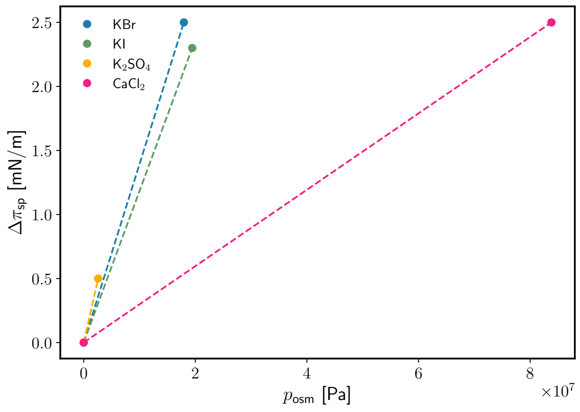

(d) Ethyl palmitate

Figure S2: Change of the equilibrium spreading pressure as a function of the osmotic pressure of the electrolyte solution. The points are experimentally determined; the dashed lines are an interpolation with a monome or binome.

The index LE designates the OA phase as liquid-expanded, as opposed to the liquid-condensed (LC) phase present in EP. At the lowest density there are some points that are likely remnants from the gaseous phase (G). Those were determined by eye and removed from consideration. Similarly, all points determined to correspond to a collapsed monolayer are removed. The fitting function is so defined, as to go through the point (S_{ref}, π_{ref}). In this case that point is (S_{sp}, π_{sp}). The spreading pressures were calculated using the interpolated functions from 2.4. The molecular area at spreading pressure is extrapolated by extending a line, passing to through the last two or three points before collapse, to the spreading pressure (see the discussion in main article). The final fitted parameters are presented on table S4. A graphical

Table S3: Fitting parameters for the dependence of the change of the equilibrium spreading pressure as a function of the osmotic pressure of the electrolyte solution.

Surfactant	Electrolyte	a_2 [Pa^{-2}]	$a_{2,err}$ [Pa^{-2}]	a_1 [Pa^{-1}]	$a_{1,err}$ [Pa^{-1}]	s [mN/m]
Oleic acid	LiCl	9.68e-17	2.86e-17	5.46e-08	6.11e-09	0.650
	NaCl	9.13e-16	3.69e-16	1.19e-07	9.08e-09	0.129
	KCl	0	0	1.84e-07	3.91e-09	0.123
	RbCl	0	0	1.97e-07	3.26e-09	0.043
	NH_4Cl	0	0	1.58e-07	2.22e-09	0.073
	KBr	-1.53e-15	8.31e-16	2.47e-07	1.50e-08	0.110
	$MgCl_2$	0	0	9.36e-08	1.40e-09	0.245
	$CaCl_2$	9.10e-17	2.61e-17	1.12e-07	2.99e-09	0.176
	$BaCl_2$	0	0	2.10e-07	4.10e-09	0.072
Diethyl sebacate	KF	0	0	2.42e-09	6.19e-10	0.016
	LiCl	0	0	8.87e-08	2.40e-09	0.107
	NaCl	0	0	8.28e-08	2.39e-09	0.085
	KCl	0	0	2.68e-08	3.87e-09	0.062
	RbCl	0	0	7.21e-09	0	0
	KBr	0	0	1.24e-07	0	0
	KI	0	0	1.61e-07	3.07e-09	0.044
	KSCN	-2.82e-15	2.75e-17	2.81e-07	5.41e-10	0.003
Ethyl palmitate	KBr	0	0	1.39e-07	0	0
	KI	0	0	1.18e-07	0	0
	K_2SO_4	0	0	1.93e-07	0	0
	$CaCl_2$	0	0	2.98e-08	0	0

comparison of the fits it presented in figure S3.

The EP data are harder to interpolate as there is a phase transition around 10 mN/m. At equilibrium the phase transition should be described by horizontal line connecting the LE and LC phases. However, the interactions between the domains in the heterogeneous region (Het) determine slower kinetics of compression.[S16,S17] As such, the measured points between LE and LC are rarely close enough to equilibrium. Here the Het region is fitted with a 4th degree polynomial

$$S_{Het}(\pi) = c_4\pi^4 + c_5\pi^3 + c_6\pi^2 + c_7\pi + c_8 \tag{S11}$$

In the final reconstruction of the isotherms the phase transition region is replaced by a horizontal line $\pi = \pi_{pt}$. Furthermore, the dynamics of the Het region also effects the first

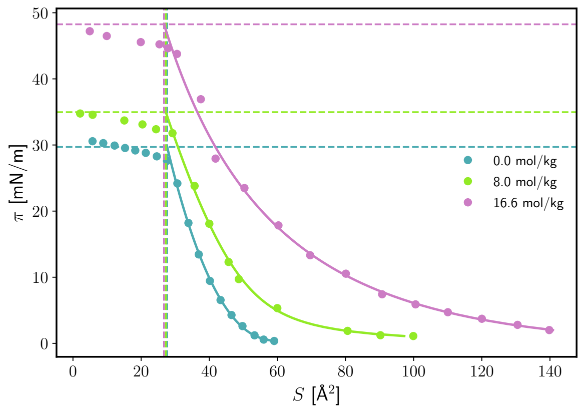

(a) LiCl

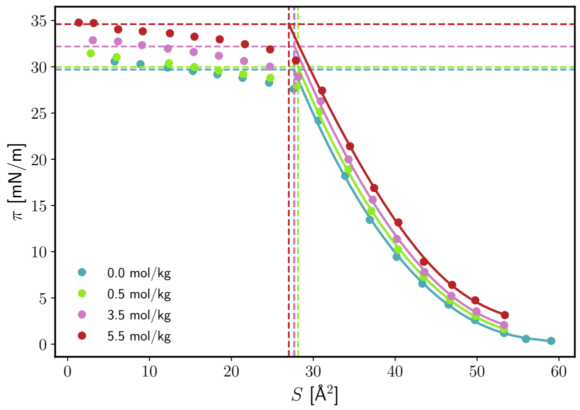

(b) NaCl

Figure S3: Surface pressure to area isotherms for OA on electrolyte solutions. The points are experimentally determined; the solid lines are a fit to the points; the dashed line define the state of the monolayer at equilibrium spread conditions.

Table S4: Fitting parameters for the dependence of the surface pressure of OA monolayers as a function of the area per surfactant molecule.

Electrolyte	$C_{\mathrm{el,m}}$ [mol/kg]	c_1 [Å^2]	$c_{1,\mathrm{err}}$ [Å^2]	c_2 [Å^2]	$c_{2,\mathrm{err}}$ [Å^2]	c_3 [Å^2]	$c_{3,\mathrm{err}}$ [Å^2]	s_{LE} [Å^2]
Water	0	13.788	0.380	2.564	0.272	0.234	0.045	0.339
LiCl	8.00	22.637	3.263	6.063	3.076	1.555	0.650	2.010
	16.60	28.858	2.253	-5.004	2.115	-0.879	0.476	1.559
NaCl	0.50	14.156	0.355	2.742	0.372	0.296	0.091	0.189
	3.50	15.286	0.407	3.482	0.447	0.484	0.116	0.207
	5.50	17.976	0.584	5.555	0.731	1.112	0.217	0.263

points in the LC region.[S16,S17] Thus, only the last two points of the isotherm are used to describe the LC region. They were fitted with a straight line

$$\mathrm{S}_{\mathrm{LC}}\left(\pi\right) = S_{\mathrm{sp}} + c_9\left(1 - \frac{\pi}{\pi_{\mathrm{sp}}}\right) \tag{S12}$$

The line is then extended to intersect with π_{pt}. The function is once again forced to go thought the point (S_{sp}, π_{sp}). The general procedure is as follows:

1. The points visually determined bellow the phase transition region are fitted to Equation S10 with $\pi_{\mathrm{ref}} = \pi_{\mathrm{sp}}$ and S_{ref} as a free parameter.

2. The highest two points are fitted to a straight line (equation S12).

3. The data points left in-between are fitted to a polynomial of the fourth degree (equation S11).

4. The intersection point between the fit for the LE region and the Het region is determined as the phase transition pressure π_{pt} and area S_{pt}.

5. The data point division for the fitting procedures are checked with regards to π_{pt} and if inconsistencies arise items 1 to 5 are redone.

6. The LE region is refitted with Equation S10 and $(S_{\text{ref}}, \pi_{\text{ref}}) = (S_{\text{pt}}, \pi_{\text{pt}})$.

The final fitting parameters are tabulated in table S5. Figure S4 shows a graphical comparison of the final fits with the experimental data.

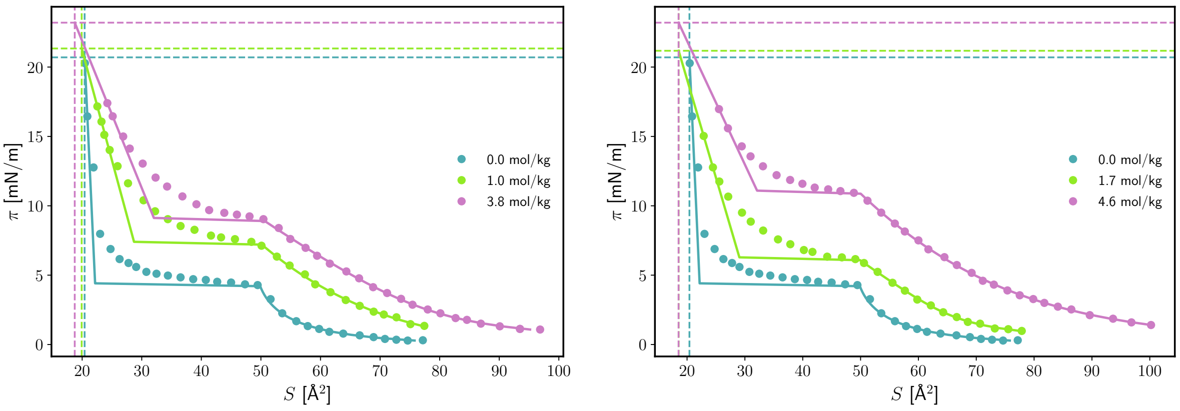

(a) KBr (b) $CaCl_2$

Figure S4: Surface pressure to area isotherms for EP on electrolyte solutions. The points are experimentally determined; the solid lines are a fit to the points; the dashed line define the state of the monolayer at equilibrium spread conditions.

Table S5: Fitting parameters for the dependence of the surface pressure of EP monolayers as a function of the area per surfactant molecule.

		LE							LC		
Electrolyte	$C_{\text{el,m}}$ [mol/kg]	c_1 [Å^2]	$c_{1,\text{err}}$ [Å^2]	c_2 [Å^2]	$c_{2,\text{err}}$ [Å^2]	c_3 [Å^2]	$c_{3,\text{err}}$ [Å^2]	s_{LE} [Å^2]	c_9 [Å^2]	$c_{9,\text{err}}$ [Å^2]	s_{LC}[Å^2]
Water	0	3.717	1.847	-3.525	1.984	-0.494	0.507	0.943	2.238	2e-13	4e-14
KBr	1.04	19.649	1.304	2.471	2.280	0.206	0.950	0.476	13.449	2e-14	5e-15
	3.77	31.546	0.986	11.947	1.391	3.334	0.464	0.573	21.894	<1e-16	<1e-16
$CaCl_2$	1.73	20.856	1.240	9.363	2.027	3.417	0.788	0.470	14.932	2e-14	8e-15
	4.56	27.286	0.554	3.201	0.831	0.913	0.296	0.315	26.014	8e-15	4e-15

Formulas S10, S11 and S12 can be analytically integrated to calculate the chemical potentials. When we account for the different phases equation 23 becomes

$$\begin{aligned}\Delta\mu_{\mathrm{OA}} =&(2c_2 - c_1 - 6c_3)\ln\left(\frac{\pi}{\pi_{\mathrm{sp}}}\right)\pi - (c_2 - 3c_3)\ln^2\left(\frac{\pi}{\pi_{\mathrm{sp}}}\right)\pi - c_3\ln^3\left(\frac{\pi}{\pi_{\mathrm{sp}}}\right)\pi \\ &+ (S_{\mathrm{sp}} + c_1 - 2c_2 + 6c_3)(\pi - \pi_{\mathrm{sp}})\end{aligned} \tag{S13}$$

$$\begin{aligned}\Delta\mu_{\mathrm{EP}} =&(2c_2 - c_1 - 6c_3)\ln\left(\frac{\pi}{\pi_{\mathrm{pt}}}\right)\pi - (c_2 - 3c_3)\ln^2\left(\frac{\pi}{\pi_{\mathrm{pt}}}\right)\pi - c_3\ln^3\left(\frac{\pi}{\pi_{\mathrm{pt}}}\right)\pi \\ &+ (S_{\mathrm{pt}} + c_1 - 2c_2 + 6c_3)(\pi - \pi_{\mathrm{pt}}) + S_{\mathrm{sp}}(\pi - \pi_{\mathrm{sp}}) + c_9\left(\pi - \frac{\pi_{\mathrm{sp}}}{2} - \frac{\pi^2}{2\pi_{\mathrm{sp}}}\right)\end{aligned} \tag{S14}$$

for OA ans EP respectfully. The resulting potentials as a function of the surface pressure can be seen in figure S5.

S2.6 Differentiation of $\Delta\mu_{\mathrm{s}}$

Unfortunately, formulae S13 and S14 can not be differentiated analytically with respect to the electrolyte concentration. In order to do that the parameters c_i's functional dependence on the concentration/osmotic pressure needs to be determined. The current data is too sparse for that. Thus, $\Delta\mu_{\mathrm{s}}$ was calculated from each isotherm and then differentiated numerically using second order (three point) central finite difference. The two phases of EP further complicate the differentiation. To calculate $(\partial\Delta\pi/\partial p_{\mathrm{osm}})_{\mu_{\mathrm{s}}}$ or $(\partial\Delta\mu_{\mathrm{s}}/\partial p_{\mathrm{osm}})_{\pi}$ the surfactant should be in the same phase at all three concentrations. This is illustrated on S6. The dashed lines confine the allowed differentiation regions. Once the proper differentiable range is determined and $(\partial\Delta\pi/\partial p_{\mathrm{osm}})_{\mu_{\mathrm{s}}}$ and $(\partial\Delta\mu_{\mathrm{s}}/\partial p_{\mathrm{osm}})_{\pi}$ are calculated numerically, the monolayer-induced adsorption of electrolyte $\Delta\Gamma_{\mathrm{el}}$ can be calculated using equations 19 and 24. The final results are presented on figure 11.

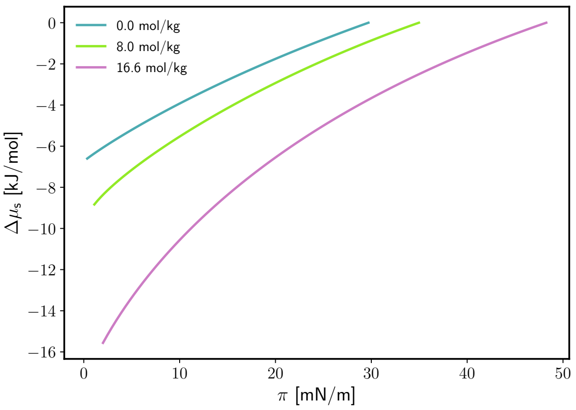

(a) Oleic acid/LiCl

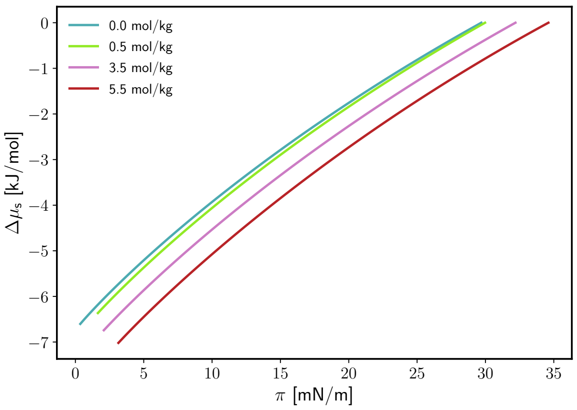

(b) Oleic acid/NaCl

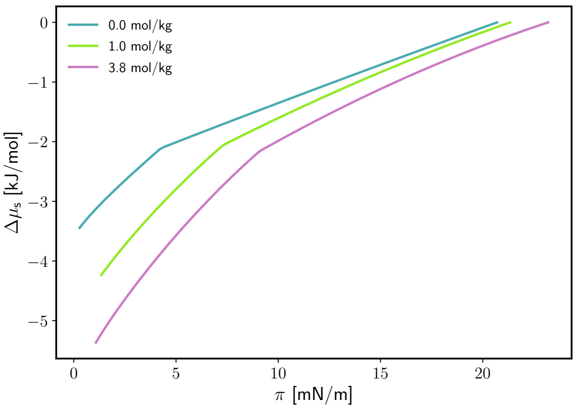

(c) Ethyl palmitate/KBr

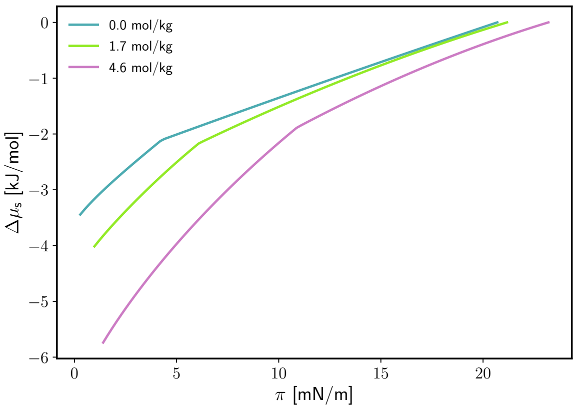

(d) Ethyl palmitate/$CaCl_2$

Figure S5: The change of surfactant chemical potential in the monolayer as a function of the surface pressure.

S3 Ancillary derivations

S3.1 Adsorption of H^+ at oleic acid monolayers

Oleic acid (HOa) at W|A dissociates leading to charging of the monolayer.

$$\mathrm{HOa} \longrightarrow \mathrm{H}^+ + \mathrm{Oa}^-$$

The equilibrium condition is

$$K_{\mathrm{HOa}} = \frac{C_{\mathrm{H}^+}\mathit{\Gamma}_{\mathrm{Oa}^-}}{\mathit{\Gamma}_{\mathrm{HOa}}} \mathrm{e}^{-e\phi^{\mathrm{S}}/kT}, \tag{S15}$$

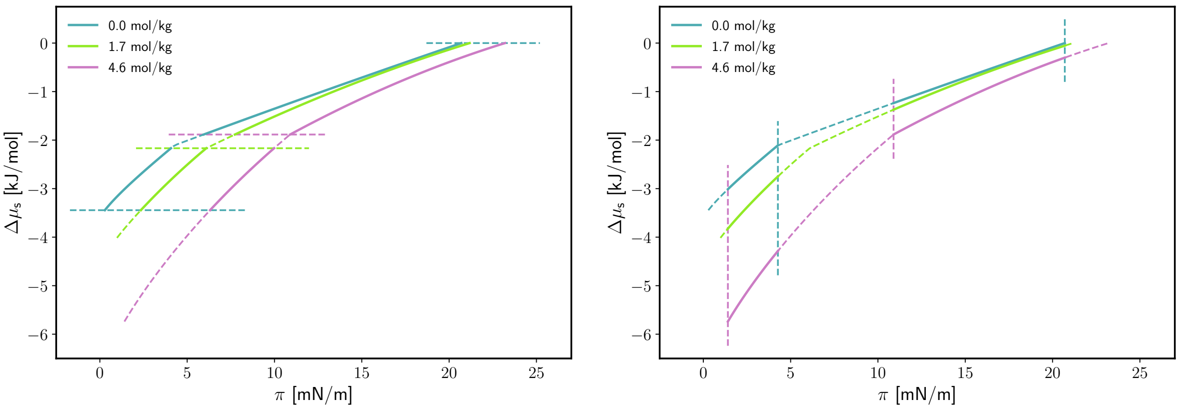

(a) Constant chemical potential

(b) Constant surface pressure

Figure S6: The change of EP chemical potential in the monolayer as a function of the surface pressure in the presence of $CaCl_2$. The solid lines define the differentiable regions.

where $C_{\mathrm{H^+}} = 10^{-2}$ M is the bulk concentration of the protons, and ϕ^{S} is the surface charge. We will assume that the surface acid dissociation constant of oleic acid K_{HOa} is equal to the bulk one (approx. 10^{-5} M). $\mathit{\Gamma}_{\mathrm{Oa^-}}$ and $\mathit{\Gamma}_{\mathrm{HOa}}$ are the surface concentrations of the dissociated and undissociated oleic acid, respectively. They are related trough the mass balance:

$$\mathit{\Gamma}_{\mathrm{HOa,t}} = \mathit{\Gamma}_{\mathrm{Oa^-}} + \mathit{\Gamma}_{\mathrm{HOa}}, \tag{S16}$$

where $\mathit{\Gamma}_{\mathrm{HOa,t}}$ is the total adsorption of oleic acid. Furthermore, the surface charge $e\mathit{\Gamma}_{\mathrm{Oa^-}}$ and potential are related as

$$\frac{N_{\mathrm{a}} e^2 \mathit{\Gamma}^2_{\mathrm{Oa^-}}}{2\varepsilon k T C_{\mathrm{H^+}}} = \mathrm{e}^{-e\phi^{\mathrm{S}}/kT} + \mathrm{e}^{e\phi^{\mathrm{S}}/kT} - 2 \tag{S17}$$

Equations S15, S16 and S17 can be solved numerically to find the three unknowns $\mathit{\Gamma}_{\mathrm{Oa^-}}$, $\mathit{\Gamma}_{\mathrm{HOa}}$ and ϕ^{S}. At monolayer density $\mathit{\Gamma}_{\mathrm{HOa,t}} = 1/25$ Å^{-2} (dense monolayer) and 25°C, only about 0.1% of the monolayer is dissociated. This gives rise to −0.5 mV surface potential and 0.004 nm^{-2} adsorption of H^+. For context, for most electrolytes, in the concentration range we are interested in, the electrolyte adsorptions on W|OA is in the order of e.g. −0.5 nm^{-2}.

S3.2 Excess size of the depletion layer

Provided the depletion and diffuse layer do not overlap, one can divide the ion surface excess Γ_i into two parts: non-electrostatic contribution Γ_i^{sp}, due to the image potential $u_{\mathrm{im},i}$ and an ion-specific interaction potential u_i acting on the i-th ion, and diffuse ion layer contribution Γ_i^{diff}, due to the charging of the interface as a result of Γ_i^{sp}. Within the linear Gouy theory it can be shown that the diffusive contributions cancel out from the electrolyte excess, i.e. Γ_{el} is determined from the non-electrostatic interactions only, $\Gamma_{\mathrm{el}} = \sum_i \Gamma_i^{\mathrm{sp}} / \sum_i \nu_i$.[S18] Moreover, at concentrations above 0.3 M, the image forces can be neglected due to electrostatic screening (Aveyard's approximation). If we finally assume that the interaction potential u_i is concentration independent, the negative slope $R_{\mathrm{el}}^* \equiv -\partial \Delta \Gamma_{\mathrm{el}}^{\epsilon} / \partial C_{\mathrm{el,M}}$ (see figure 7) can be found as:

$$R_{\mathrm{el}}^* = -\frac{1}{\nu} \sum_i \nu_i \int_0^{\infty} (\mathrm{e}^{-u_i^{\mathrm{W|M}}/kT} - \mathrm{e}^{-u_i^{\mathrm{W|A}}/kT})\, \mathrm{d}z. \tag{S18}$$

The integrals under the sum in equation S18 have dimension of length and can be thought of as negative excess size of the ion depletion layer R_i^* in the presence of monolayer. Therefore, the quantity R_{el}^* is the mean excess size of the electrolyte depletion layer, related to the ion characteristic R_i^* as $\nu R_{\mathrm{el}}^* = \sum_i \nu_i R_i^*$. The results in figure 7 are in agreement with the prediction of a linear slope up to concentration 2 mol/kg. At higher concentrations the interaction potential u_i seems to be concentration dependent. The calculated R_{el}^*, with $V_{\mathrm{s}} = 32$ mL/mol, are presented in table S6.

Table S6: The initial negative slopes $\partial \Delta \Gamma_{\mathrm{el}}^{\epsilon} / \partial C_{\mathrm{el,M}}$ for different electrolytes on equilibrium spread monolayer of OA calculated with $V_{\mathrm{s}} = 32$ mL/mol. The value of R_{el}^* compares the affinity of the electrolyte to W|M with that to W|A.

Electrolyte	LiCl	NaCl	KCl	RbCl	NH_4Cl	KBr	$MgCl_2$	$CaCl_2$	$BaCl_2$
R_{el}^* [Å]	1.370	0.672	0.071	-0.069	0.325	-0.473	0.984	0.792	-0.197

S3.3 Depletion layer thickness and surfactant partial molar volume in water

S3.3.1 Plane of ε discontinuity

In the MS and similar models the W|A surface is defined as the plane of discontinuity of the medium properties. When it comes to the image forces the relevant property is the dielectric permittivity ε of the medium. It is represented as a stepwise function from ε_0 in the air phase to $\varepsilon_{\mathrm{w}} \approx 78\epsilon_0$ in the water phase (see figure S7b). It has been experimentally found that the distance between the plane of ε discontinuity and the average position of the outside edge of the uppermost layer of water molecules, i.e. the hydrophobic gap, is approximately one effective water molecule radius. Thus, to ensure agreement between the theory and the experiment, the plane of ε discontinuity is positioned in the middle of the uppermost layer of water molecules[S18,S19] (see figure S7a). The position of the plane of ε discontinuity is relevant not only for the image forces acting on the dissolved ions, but also tightly related to the immersion of a surfactant molecule. An amphiphile molecule can be divided into polar and apolar segments (head and tail). The dividing point between them we call the hydrophilic-lyophilic centre (HL centre). Here we make the assumption that, when introducing a surfactant molecule in the system, the HL centre positions itself on the plane of ε discontinuity. However, the addition of the surfactant changes the ε profile and offsets the plane of ε discontinuity, thus indirectly effecting the electrolyte. In this section we are interested in roughly estimating this shift.

Fist of all, we neglect any correction on the position of the surfactant molecules themselves. We assume, the average position of the HL centre is the same for diluted and concentrated monolayers and defined by the plane of ε discontinuity in the infinitely diluted region, i.e. on the neat W|A interface, which we define as the origin of the system. In order to keep the simple stepwise approximation for the ε profile, we introduce two intermediary layers with depth independant dielectric permittivities (see figure S7d). One is a mixture of surfactant tails and air and the other is a mixture of surfactant heads and water. Fur-

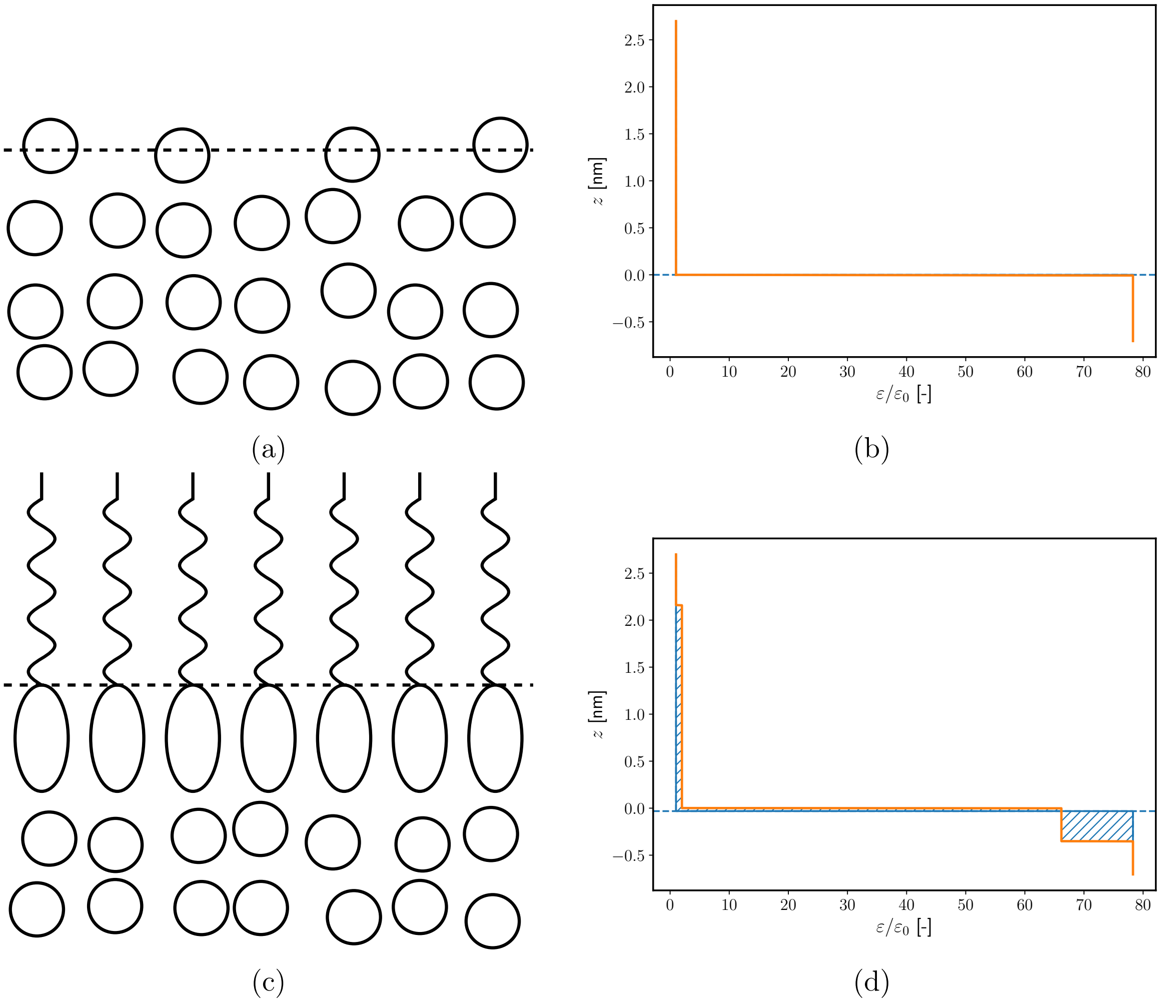

Figure S7: (a and c) schematic representation of the structure of the interface W|A and W|M, respectively. (b and d) approximated stepwise ε profile through W|A and W|M, respectively.

thermore, we assume that each segment has the same dielectric response as a bulk phase of the closest substance, e.g. the head of an aliphatic acid behaves as formic acid $\varepsilon \approx 58\varepsilon_0$. Thus, defining the ε profile boils down to finding the the dielectric permittivities of the two intermediary layers. That can be done with the Clausius–Mossotti relation. However, in the interest of simplicity and brevity, we will instead assume a linear relationship between ε and the concentrations:

$$\epsilon = \epsilon_0 + \sum_i \frac{\alpha_i}{N_\mathrm{A}} C_i, \tag{S19}$$

where α_i is the molecular polarizability of the $i^{\text{-th}}$ component and the summation is over all constituents. Using equation S19 one can express the polarizabilities α_i through the dielectric permittivity ε_i^∞ and molar volume V_i^∞ of the pure substance, e.g. for alkane $\alpha_\mathrm{alkane} = \left(\epsilon_\mathrm{alkane}^\infty - \epsilon_0\right) N_\mathrm{A} V_\mathrm{alkane}^\infty$. Thus, we can find the dielectric permittivities of the head and tail layers as:

$$\epsilon_\mathrm{tail} = \epsilon_0 + \left(\epsilon_\mathrm{tail}^\infty - \epsilon_0\right) C_\mathrm{tail} V_\mathrm{tail}^\infty \tag{S20}$$

$$\epsilon_\mathrm{head} = \epsilon_\mathrm{w} + \left(\epsilon_\mathrm{head}^\infty - \epsilon_\mathrm{w}\right) C_\mathrm{head} V_\mathrm{head}^\infty, \tag{S21}$$

where we used the condition $\sum_i C_i(z) V_i = 1$ to remove the concentration of water in the head layer. Here C_tail and C_head are the concentrations of tails and heads in their respective layers.

The plane of ε discontinuity is located at some distance d from the origin (see figure S8). Since the plane of ε discontinuity corresponds to the plane of ε zero excess, the following condition holds:

$$\int_{-\infty}^{-d} \left(\epsilon(z) - \epsilon_\mathrm{w}\right) \mathrm{d}z + \int_{-d}^{\infty} \left(\epsilon(z) - \epsilon_0\right) \mathrm{d}z = 0 \tag{S22}$$

Within the current model, the last equation can be restated as:

$$\int_{-\infty}^{0} \left(\epsilon_\mathrm{head} - \epsilon_\mathrm{w}\right) \mathrm{d}z + \int_{0}^{\infty} \left(\epsilon_\mathrm{tail} - \epsilon_0\right) \mathrm{d}z = -\left(\epsilon_\mathrm{w} - \epsilon_0\right) d \tag{S23}$$

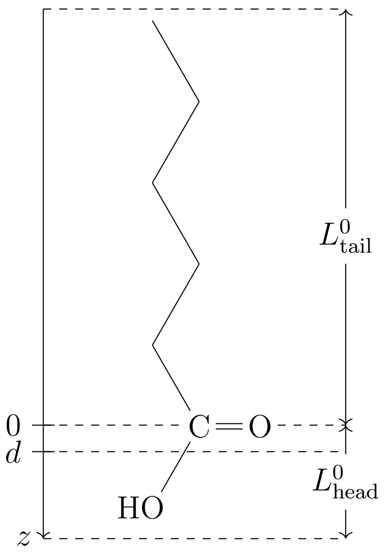

Figure S8: Geometry of a carboxylic surfactant

Combining that with equations S20 and S21 gives an explicit solution for the shift of the plane of ε discontinuity:

$$d = -\frac{\epsilon_{\mathrm{head}}^{\infty} - \epsilon_{\mathrm{w}}}{\epsilon_{\mathrm{w}} - \epsilon_0} \mathit{\Gamma}_{\mathrm{s}} V_{\mathrm{head}}^{\infty} - \frac{\epsilon_{\mathrm{tail}}^{\infty} - \epsilon_0}{\epsilon_{\mathrm{w}} - \epsilon_0} \mathit{\Gamma}_{\mathrm{s}} V_{\mathrm{tail}}^{\infty} \tag{S24}$$

As it can be seen within these rough approximations the shift of the plane of ε discontinuity is a linear function of the surfactant adsorption $\mathit{\Gamma}_{\mathrm{s}}$. The closer the segments are to the two respective mediums in terms of dielectric permittivities, the smaller d. The maximum possible shift is at maximum monolayer density, which we assume is the equilibrium spread monolayer $\mathit{\Gamma}_{\mathrm{s,sp}}$. The product $\mathit{\Gamma}_{\mathrm{s,sp}} V_{\mathrm{i}}^{\infty}$ is simply the characteristic length of the segment, e.g. $\mathit{\Gamma}_{\mathrm{s,sp}} V_{\text{formic acid}}^{\infty} \approx 3.5$ Å. Furthermore, the fraction $(\epsilon_{\mathrm{i}}^{\infty} - \epsilon_{\mathrm{j}}) / (\epsilon_{\mathrm{w}} - \epsilon_0)$ is generally small. The resulting shift d_{sp} is about an order of magnitude lower than the effective water radius R_{w}, e.g. for OA $d_{\mathrm{sp}} \approx 0.3$ Å (see figure S7d). Thus we can conclude, that any effect coming from the offset of the plane of ε discontinuity is quite small.

S3.3.2 Depletion layer thickness

Within the MS model the work required for an ion to penetrate into the topmost layer of molecules is assumed infinite. Thus, for a monovalent ion i on W|A the thickness of the depletion layer R_i is simply (see figure S7a)

$$R_i = R_{0,i} + R_{\mathrm{w}}. \tag{S25}$$

This simple assumption works incredibly well and allows for the qualitative prediction of the surface tension of a large set of systems. When a dense monolayer is spread on the surface, the topmost layer is now of surfactant molecules, that have a different size. Taking into account that R_i is defined in relation to the plane of ε discontinuity, it can be found as (see figure S7c)

$$R_i = R_{0,i} + L^0_{\mathrm{head}} - d. \tag{S26}$$

The correction d is small. On the other hand, L^0_{head} could be quite larger than R_{w}, e.g. for OA L^0_{head} is approximately 3.5 Å compared to $R_{\mathrm{w}} = 1.4$ Å. The difference $\Delta R_i \equiv L^0_{\mathrm{head}} - R_{\mathrm{w}} \sim 2$ Å results in monolayer-induced adsorption $\Delta\mathit{\Gamma}_{\mathrm{i}} \sim -\Delta R_i C_i \sim -0.12\ \mathrm{nm}^{-2}$, which is comparable with the $\Delta\mathit{\Gamma}_{\mathrm{el}}$ calculated above.

For a monolayer of intermediate densities, some ions will be underneath water molecules and some underneath surfactant molecules. Therefore, R_i is a weighted averaged value. If we assume there is no specific interaction between the ions and surfactant molecules, the thicknesses are simply wighted by the surface coverage $\theta = \mathit{\Gamma}_{\mathrm{s}}/\mathit{\Gamma}_{\mathrm{s,sp}}$

$$R_{i,av} = R_{0,i} + R_{\mathrm{w}}\,(1-\theta) + \left(L^0_{\mathrm{head}} - d\right)\theta. \tag{S27}$$

Note that $R_{i,av}$ is also a linear function of $\mathit{\Gamma}_{\mathrm{s}}$. Thus, the larger size of the surfactant molecule on its own can not explain the complicated relationship between $\Delta\mathit{\Gamma}_{\mathrm{el}}$ and $\mathit{\Gamma}_{\mathrm{s}}$ presented in figure 11.

S3.3.3 Partial molar volume

Lets find the volume V_{Md} between an arbitrary plane M (see figure S9) and the plane of ε discontinuity. Only a part of the surfactant molecule is inside the slice Md. That part we characterise with a volume $V_\mathrm{s}(d)$. Then, the volume the surfactant molecules occupy in the slice Md is $V_\mathrm{s}(d)n_\mathrm{s}$, where n_s is the number of molecules on the surface. Applying the same logic for the water molecules, we can express V_{Md} as

$$V_{Md} = V_\mathrm{s}(d)n_\mathrm{s}^{Md} + V_\mathrm{w}(d)n_\mathrm{w}^{Md}. \tag{S28}$$

This is equivalent to the condition 14. Dividing both sides by V_{Md}, we get

$$1 = V_\mathrm{s}(d)C_\mathrm{s}^{Md} + V_\mathrm{w}(d)C_\mathrm{w}^{Md}, \tag{S29}$$

where C_s^{Md} and C_w^{Md} refer to the concentrations in the surface layer Md.

At intermediate surface coverage the surface layer is a mixture of surfactant and water molecules. The model presented here can not distinguish between a homogeneous mixture and a segregated mixture (see figures S9a and S9b). The average shift d_av in figure S9b corresponds to the d in figure S9a. It can easily be seen that if one averages the shift of the plane of ε discontinuity in figure S9b, weighted by the surface coverage, one will get formula S24; $d_\mathrm{av}(\mathit{\Gamma}_\mathrm{s}) = d_\mathrm{sp}\theta(\mathit{\Gamma}_\mathrm{s})$. A similar logic applies to the volume V_{Md}. In a segregated system the volume V_{Md} with respect to the average d is a sum of the volumes of each domain; e.g. the orange area in figure S9a is equal to the sum of the magenta and cyan areas in figure S9b. This allows us to write

$$1 = V_\mathrm{s}(d_\mathrm{sp})C_\mathrm{s}^{Md} + V_\mathrm{w}(0)C_\mathrm{w}^{Md}. \tag{S30}$$

Here $V_\mathrm{w}(0)$ is a constant, characteristic of water. We assume that $V_\mathrm{w}(0)$ is equal to the bulk molecular volume of water molecules. For the latter we know that $V_\mathrm{w}C_\mathrm{w} = 1$. If we subtract

that from equation S30 and integrate over the depth z, we get

$$V_\mathrm{s}(d_\mathrm{sp})\mathit{\Gamma}_\mathrm{s}^\varepsilon + V_\mathrm{w}\mathit{\Gamma}_\mathrm{w}^\varepsilon = 0. \tag{S31}$$

This is a version of equation 15 in the absence of electrolyte. The surfactant molar volume V_s that appears in equation 15 and all that follow it is defined by this derivation as the volume of a surfactant molecule penetrating beneath the plane of ε discontinuity at maximum surface density. It can be found as the depth of penetration $L^0_\mathrm{head} - d_\mathrm{sp}$ times the cross-sectional area of the head group. If we assume the cross-sectional area of the carboxylic group is 16.5 Å^2, the molar volume of OA is approximately 32 mL/mol. That is just below the molar volume of formic acid (35 mL/mol).

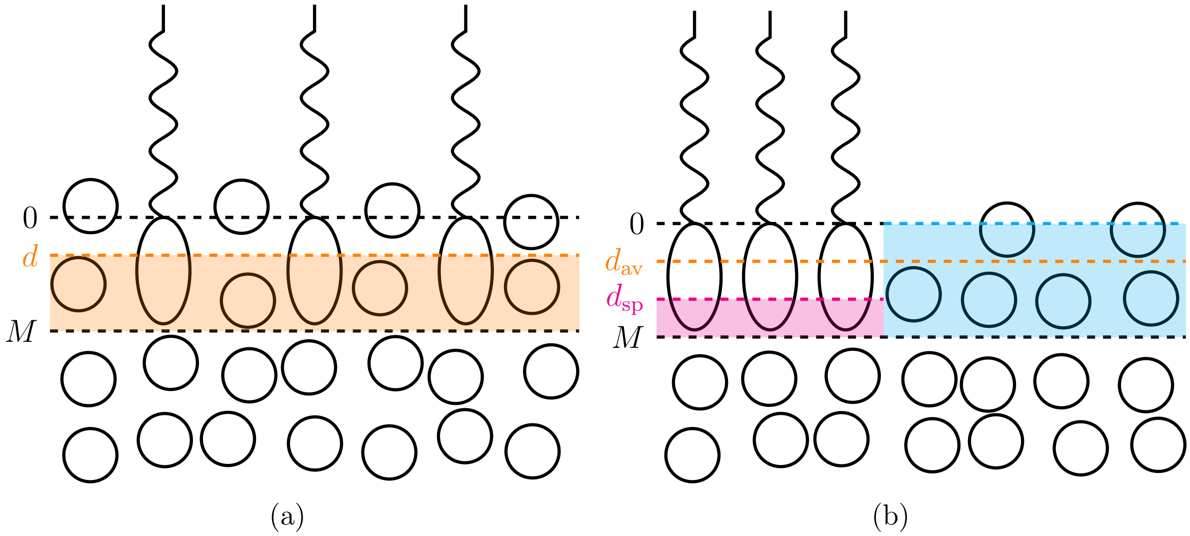

Figure S9: Schematic representation of the structure of the interface W|M at intermediate surface coverage. (a) homogeneous surface; (b) segregated surface.

References

(S1) Harned, H. S.; Owen, B. B.; King, C. The physical chemistry of electrolytic solutions. *Journal of The Electrochemical Society* **1959**, *106*, 15C.

(S2) Hamer, W. J.; Wu, Y.-C. Osmotic coefficients and mean activity coefficients of uni-univalent electrolytes in water at 25° C. *Journal of Physical and Chemical Reference Data* **1972**, *1*, 1047–1100.

(S3) Barthel, J. M.; Krienke, H.; Kunz, W. *Physical chemistry of electrolyte solutions: modern aspects*; Springer Science & Business Media, 1998; Vol. 5.

(S4) Buchner, R.; Hefter, G. T.; May, P. M. Dielectric relaxation of aqueous NaCl solutions. *The Journal of Physical Chemistry A* **1999**, *103*, 1–9.

(S5) Harris, F. E.; O'Konski, C. T. Dielectric properties of aqueous ionic solutions at microwave frequencies. *The journal of physical chemistry* **1957**, *61*, 310–319.

(S6) Chen, T.; Hefter, G.; Buchner, R. Dielectric spectroscopy of aqueous solutions of KCl and CsCl. *The Journal of Physical Chemistry A* **2003**, *107*, 4025–4031.

(S7) Hasted, J.; Ritson, D.; Collie, C. Dielectric properties of aqueous ionic solutions. Parts I and II. *The Journal of Chemical Physics* **1948**, *16*, 1–21.

(S8) Marcus, Y. Ion Properties, Marcus Dekker. *Inc, New York* **1997**,

(S9) Robinson, R.; Stokes, R. Electrolyte Solutions Butterworth & Co.(publ.) Ltd. **1959**,

(S10) Stokes, R. A thermodynamic study of bivalent metal halides in aqueous solution. Part XIII. Properties of calcium chloride solutions up to high concentrations at 25°. *Transactions of the Faraday Society* **1945**, *41*, 637–641.

(S11) Gilby, A.; Heymann, E. Oleic Acid Monolayers on Concentrated Salt Solutions. *Australian Journal of Chemistry* **1952**, *5*, 160.

(S12) Pankratov, A. Properties of monomolecular layers on solutions of salts I. *Acta Physicochim. URSS* **1939**, *10*, 45–54.

(S13) Frumkin, A.; Pankratov, A. Properties of monomolecular layers on solutions of salts II. *Acta Physicochim. URSS* **1939**, *10*, 55–64.

(S14) Donnison, J. A.; Heymann, E. The equilibrium spreading pressure of oleic acid and of ethyl sebacate on concentrated salt solutions. *Transactions of the Faraday Society* **1946**, *42*, 1–5.

(S15) Hamer, W. J.; Wu, Y. Osmotic Coefficients and Mean Activity Coefficients of Uni-univalent Electrolytes in Water at 25°C. *Journal of Physical and Chemical Reference Data* **1972**, *1*, 1047–1100.

(S16) Peshkova, T. V.; Minkov, I. L.; Tsekov, R.; Slavchov, R. I. Adsorption of ions at uncharged insoluble monolayers. *Langmuir* **2016**, *32*, 8858–8871.

(S17) Minkov, I. L.; Arabadzhieva, D.; Salama, I. E.; Mileva, E.; Slavchov, R. I. Barrier kinetics of adsorption–desorption of alcohol monolayers on water under constant surface tension. *Soft matter* **2019**, *15*, 1730–1746.

(S18) Slavchov, R. I.; Novev, J. K.; Peshkova, T. V.; Grozev, N. A. Surface tension and surface $\Delta\chi$-potential of concentrated Z+: Z- electrolyte solutions. *Journal of colloid and interface science* **2013**, *403*, 113–126.

(S19) Slavchov, R. I.; Novev, J. K. Surface tension of concentrated electrolyte solutions. *Journal of colloid and interface science* **2012**, *387*, 234–243.
